# The Neuroprotective Functions of Transforming Growth Factor Beta Proteins

**DOI:** 10.3390/ijms13078219

**Published:** 2012-07-03

**Authors:** Arpád Dobolyi, Csilla Vincze, Gabriella Pál, Gábor Lovas

**Affiliations:** 1 Department of Anatomy, Histology and Embryology, Semmelweis University, Tuzolto u. 58, Budapest H-1094, Hungary; E-Mails: csilla.vincze@gmail.com (C.V.); pgabi4@gmail.com (G.P.); 2 Neuromorphological and Neuroendocrine Research Laboratory, Hungarian Academy of Sciences, H-1025 Budapest, Hungary; 3 Department of Neurology, Semmelweis University, Balassa u. 6, Budapest H-1083, Hungary; E-Mail: lovasgab@hotmail.com; 4 Department of Neurology, Jahn Ferenc Teaching Hospital, Köves u. 2-4, Budapest H-1204, Hungary

**Keywords:** TGF-β, stroke, lesion, brain injury, neurodegeneration, neuroprotection

## Abstract

Transforming growth factor beta (TGF-β) proteins are multifunctional cytokines whose neural functions are increasingly recognized. The machinery of TGF-β signaling, including the serine kinase type transmembrane receptors, is present in the central nervous system. However, the 3 mammalian TGF-β subtypes have distinct distributions in the brain suggesting different neural functions. Evidence of their involvement in the development and plasticity of the nervous system as well as their functions in peripheral organs suggested that they also exhibit neuroprotective functions. Indeed, TGF-β expression is induced following a variety of types of brain tissue injury. The neuroprotective function of TGF-βs is most established following brain ischemia. Damage in experimental animal models of global and focal ischemia was shown to be attenuated by TGF-βs. In addition, support for their neuroprotective actions following trauma, sclerosis multiplex, neurodegenerative diseases, infections, and brain tumors is also accumulating. The review will also describe the potential mechanisms of neuroprotection exerted by TGF-βs including anti-inflammatory, -apoptotic, -excitotoxic actions as well as the promotion of scar formation, angiogenesis, and neuroregeneration. The participation of these mechanisms in the neuroprotective effects of TGF-βs during different brain lesions will also be discussed.

## 1. Introduction

Transforming growth factors were originally named based on their abilities to induce a transformed phenotype in non-neoplastic rat kidney fibroblasts [1]. Transforming growth factor betas (TGF-βs) belong to a group of proteins that also includes bone morphogenic factors, anti-Mullerian hormone, activins and inhibins [2]. These proteins share some structural homology but possess separate receptors and participate in different functions. TGF-βs are prominent members of this group of proteins commonly referred to as the transforming growth factor beta superfamily. TGF-βs have pleiotropic functions in various organs. They regulate the growth, differentiation and survival of different cell types [3]. Therefore, they can be considered growth factors. They also influence different cells in the immune system, and consequently, are also considered cytokines [4]. TGF-βs include three mammalian isoforms (TGF-β1, -β2, and -β3). An additional three isoforms have been identified in lower vertebrate species [5]. TGF-β subtypes are encoded by separate genes but demonstrate sequence homology and have similar mechanisms for processing and activation [6].

TGF-βs are produced as pro-TGF-βs, which form dimers. Both proteins are cleaved in the Golgi apparatus and remain non-covalently associated in the so-called small latency associated complex of TGF-βs. The complex is covalently bound to latent TGF-β binding proteins (LTBPs) in the extracellular space and forms the large latent complex of TGF-β [7]. There are 4 LTBPs identified, however, only limited information is available on their selectivity to bind TGF-βs. *In vitro*, LTBP-1 and LTBP-4 bind all three types of TGF-βs, LTBP-3 has some selectivity for TGF-β1 while LTBP-2 does not seem to bind any TGF-βs [8].

**Figure 1:**
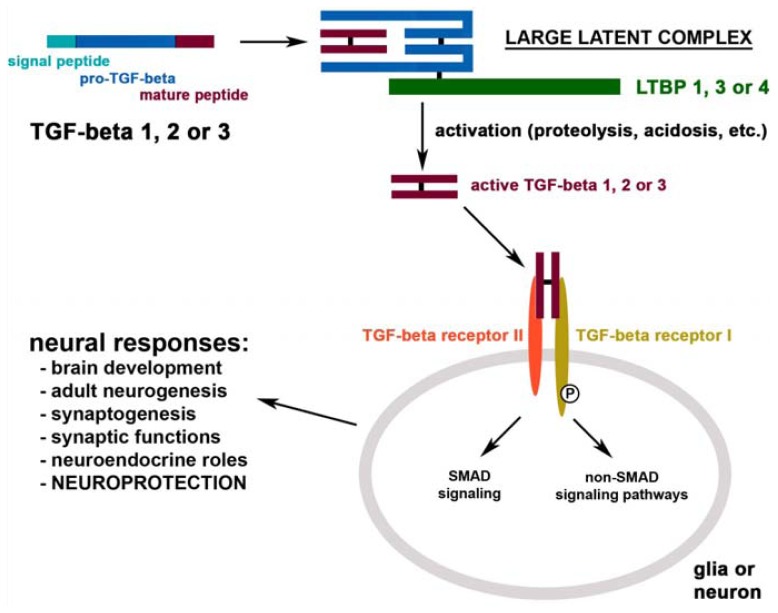
Summary diagram on the chemistry and actions of transforming growth factor beta (TGF-β)s in the nervous system.

The activation of TGF-βs requires their release from the large latent complex (Figure 1). There are several ways to induce the release TGF-βs *in vitro* including acidosis and proteolytic cleavage [9]. However, the exact physiological mechanisms remain to be determined. The free TGF-βs can travel as dimers to receptors at the extracellular surface of the target cell, and bind to heteromeric complexes of type I and II receptors, which belong to the serine/threonine kinase family of receptors. In most cells, TGF-βs signal via the canonical type I receptor TGF-β receptor I/activins-like kinase receptor 5 (Alk5). In endothelial cells and in neurons, TGF-βs may also signal via the type I receptor Alk1 [10]. The type II receptor then phosphorylates the type I receptor, which relays the signal by binding and phosphorylating a receptor-regulated Smad protein [11]. Alk5 induces phosphorylation of Smads 2 and 3 while Alk1 mediates phosphorylation of Smads 1, 5 and 8 [10]. The activated receptor-regulated Smad proteins form complexes with Smad4 [12]. Active Smad complexes translocate into the nucleus to exert their actions on gene expression [13]. TGF-βs may also use non-Smad signaling pathways including the phosphoinositide 3-kinase-Akt-mTOR pathway, the small GTPases Rho, Rac, and Cdc42, and the Ras-Erk-MAPK pathway [14].

## 2. TGF-β in the Intact Brain

### 2.1. The Distribution of TGF-βs, Their Binding Proteins and Receptors in the Brain

The distribution pattern of TGF-βs was established at the protein level by means of immunohistochemistry [15] and at the mRNA level using *in situ* hybridization histochemistry [16] as summarized in Table 1. TGF-β1 immunoreactivity was reported to be constitutively present only in meninges and the choroid plexus in the brain [15,17] while a more widespread expression of the mRNA of this subtype was described including intense labeling in some cortical and hippocampal cells, the medial preoptic area, the paraventricular hypothalamic nucleus, the central amygdaloid nucleus, and the superior olive [16]. TGF-β2 and -β3 immunoreactivities were present in distinct layers of the cerebral cortex. In addition, some hippocampal regions, as well as widely distributed cells in the hypothalamus and amygdala contained TGF-β2 and -β3. Intense labeling of these subtypes was also described in brainstem monoaminergic neurons and motor nuclei [15,16]. In turn, the striatum, most thalamic nuclei, and the superior colliculus were almost devoid of TGF-β2 and -β3 mRNA and immunoreactivities. However, considerable differences between the distribution of mRNAs and immunoreactivities of TGF-βs have also been reported. TGF-β2 and -β3 immunoreactivities entirely overlapped and, in general, were found in large multipolar neurons [15] with the level of TGF-β2 being considerably higher [18]. In some areas, including brainstem motoneurons and the area postrema, the 2 subtypes had similar mRNA expression patterns with high intensity labeling suggesting that different subtypes of TGF-βs may be co-expressed in the same cell. In most brain areas, however, the distributions of TGF-β2 and -β3 mRNAs were markedly different. In the cerebral cortex, TGF-βs were expressed in different layers. In the hippocampus, TGF-β2 was abundantly expressed only in the dentate gyrus while TGF-β3 in the CA2 region and the dentate gyrus. In the cerebellum, TGF-β2 was present in the Purkinje cell layer while TGF-β3 mRNA was absent in the cerebellum. In addition, the medial mamillary nucleus, the parafascicular thalamic nucleus and the choroid plexus expressed predominantly TGF-β2 while the reticular thalamic nucleus, the superior colliculus, and the inferior olive contained almost exclusively TGF-β3 mRNA [16].

The four subtypes of LTBPs also had distinct distribution patterns in the brain based on the localization of their mRNAs [19]. Comparison of the distribution of TGF-β and LTBP subtypes suggested that all three subtypes of TGF-βs are co-expressed with LTBP3 in the brain. In addition, TGF-βs might also bind to other types of LTBPs in certain brain regions. For example, the distribution of TGF-β1 and LTBP4 is similar in the supraoptic nucleus and the central nucleus of the amygdala. The choroid plexus, where TGF-β2 expression is dominant contains LTBP1 and 3. The inferior olive and the arcuate nucleus, brain areas with dominant TGF-β3 expression contain large amount of LTBP4 and LTBP1, respectively [19]. Nevertheless, further double labeling studies are needed to actually establish co-expression of different subtypes of TGF-βs and LTBPs in single cells of the nervous system [20].

Although the topographical distribution of TGF-β receptors in the central nervous system (CNS) has not been systematically described, the available data suggest widespread localization. When TGF-β receptor mRNA was detected by RT-PCR in rats at different stages of development similar levels were found in several regions of the CNS, including cortex, midbrain, cerebellum, brain stem and hippocampus [21].

### 2.2. The Physiological Functions of TGF-βs in the Brain

The physiological roles of TGF-βs in the nervous system are not fully understood. Their diverse roles in the development of the CNS have been established. Additional functions in neuronal transmission and neuroendocrine regulations have also been suggested recently (Figure 1).

#### 2.2.1. The Role of TGF-βs in Brain Development

During the development of the CNS, TGF-β immunolabeling was most prominent in zones where neuronal differentiation occurs and less intense in zones of active proliferation [22]. Subsequent *in vitro* experiments using quail neural crest cell demonstrated that TGF-β inhibited proliferation of neural crest cells while neurogenesis increased significantly in the presence of TGF-β [23,24]. TGF-β had an anti-mitotic effect on progenitors and increased expression of neuronal markers in hippocampal and cortical primary cell cultures of developing mouse [25]. These effects were dependent upon Smad4. TGF-β may also play a role in the regulation of adult neurogenesis as it had a pro-neurogenic effect in the dentate gyrus in a model of increased neurogenesis by adrenalectomy as well as in the subventricular zone when administered chronically with adenoviral vectors expressing TGF-β [26]. Furthermore, adrenalectomy increased TGF-β levels in the dentate gyrus while blockade of TGF-β biological activity by administration of an anti-TGF-β type II receptor antibody diminished neurogenesis [27]. Apart from playing a role in the adoption of neuronal cell fate, TGF-β may also be involved in the differentiation of selected neuronal subtypes at the expense of other subtypes. Within the intermediate and ventral domains, Smad3 promoted differentiation of ventral interneurons at the expense of motoneuron generation [28]. In turn, the survival of motoneurons may also depend on TGF-βs as a potentially continuous trophic support factor from muscle fibers or other cell types. Using cultures of purified chick embryonic motoneurons, TGF-βs acted synergistically with basic fibroblast growth factor to keep motoneurons alive [29]. Indeed, motoneurons were shown to synthesize TGF-β receptors and to transport them anterogradely, where they were inserted into the axonal membrane and nerve terminal [30]. Furthermore, TGF-β2 was detected in the synaptic portions of muscle fibers, motoneurons and in injured nerves, indicating that motoneurons may be exposed to multiple and potentially redundant sources of TGF-β2 [30]. In addition, double-ligation experiments were used to demonstrate that motoneurons transport TGF-β2 up and down their axons [30]. To test the effect of TGF-β on motoneuron survival *in vivo*, TGF-β2 was administered to the hypoglossal nucleus following the avulsion of the hypoglossal nerve in adult rats, which caused a significant attenuation of the motoneuron cell death [31]. TGF-β2 was, however, unable to prevent or reduce the axotomy-induced down regulation of choline acetyltransferase suggesting that TGF-β2 is only one of the growth factors regulating the homeostasis of motoneurons [31]. A role of TGF-β in the differentiation and survival of other, as yet unexplored neuronal cell types might also be possible. As far as glial cells, data are available that TGF-β regulates Schwann cell proliferation induced by neuronal contact in the peripheral nervous system [32,33].

The effect of TGF-β on synaptogenesis has also been proposed. In particular, TGF-β1 was identified as the molecule responsible for the synaptogenesis promoting effect of Schwann cell-conditioned medium in Xenopus nerve-muscle co-cultures [34]. TGF-β1 increased agrin expression and synaptogenesis along nerve-muscle contacts while immunodepletion of TGF-β1 with a specific antibody abolished the synaptogenic effect of Schwann cell-conditioned medium [34]. These results indicate that TGF-β1 may be a glial signal that instructs neurons to switch from a “growth state” to a “synaptogenic state”.

#### 2.2.2. TGF-βs and the Modulation of Synaptic Transmission

TGF-β2 was demonstrated to influence synaptic transmission, rather than synaptogenesis, at some central synapses [35]. TGF-β2 was found to be essential for proper synaptic function in the pre-Botzinger complex, a central rhythm organizer located in the brainstem while it was not crucial for the morphology and function of the neuromuscular junction of the diaphragm muscle. Genetic deletion of TGF-β2 in mice strongly impaired both GABA/glycinergic and glutamatergic synaptic transmission in the pre-Botzinger complex area, while numbers and morphology of central synapses of knock-out animals were indistinguishable from their wild-type littermates [35]. The role of TGF-β in synaptic transmission might be the basis of its proposed function in synaptic facilitation. Prolonged treatment with TGF-β2 induced facilitation of evoked postsynaptic currents in hippocampal neurons suggesting that it may play a role in the cascade of events underlying long-term synaptic facilitation [36].

#### 2.2.3. Proposed Neuroendocrine Functions of TGF-βs

The potential involvement of TGF-β in central reproductive regulation is also an emerging topic. Gonadotropin-releasing hormone (GnRH) neurons in the preoptic area contain TGF-β receptors as well as SMAD2/3 suggesting that they are fully capable of responding directly to TGF-β1 stimulation [37]. Subsequent double-labeling experiments showed that astrocytes in the preoptic area expressed TGF-β1 mRNA and that GnRH perikarya were often found in close association with TGF-β1 mRNA-expressing cells [38]. TGF-β1 was shown to be a neuroprotective component of astrocyte-induced medium [39,40] suggesting the ability of astrocytes to release TGF-βs. Furthermore, incubation of preoptic explants with TGF-β1 caused a significant, dose-dependent decrease in GnRH mRNA expression in individual neurons. This effect was inhibited by addition of the soluble form of TGF-β receptor II to the incubation medium [38]. These results support that astrocyte-derived TGF-β1 may directly influence GnRH expression and/or secretion *in vivo* by acting on the perikarya of GnRH neurons [20].

TGF-β1 and -β3 also co-localize with arginine vasopressin in magnocellular neurons of the supraoptic and paraventricular nuclei of the hypothalamus suggesting that TGF-β secreted by the neurohypophysis might regulate the proliferation and secretion of certain anterior pituitary cells [41]. Furthermore, a diurnal pattern of expression of TGF-β as well as SMAD3 was found in the suprachiasmatic and paraventricular nuclei of young animals, a rhythm that was not observed in older mice suggesting a diurnal and age-dependent function of the TGF-β system in these nuclei [42]. So far, the regulatory role of TGF-β on hormone secretion, gene transcription, and cellular growth of prolactin-producing cells has been shown. TGF-β inhibited the transcriptional activity of the estrogen receptor although estrogens had no effect on TGF-β specific Smad protein transcriptional activity in prolactin producing cells [43]. Interactions of TGF-β and estrogen have been demonstrated for brain cells as well. A neuroprotective action of estrogen was suggested to be mediated by TGF-β released from astrocytes [40].

These initial studies support the involvement of TGF-βs in a variety of neuroendocrine regulations. TGF-βs may interact with established neuropeptides reducing the conceptual gap between growth factors/cytokines and regulatory neuropeptides. The newly proposed neuroendocrine actions are also consistent with the widespread expression of TGF-βs in various hypothalamic centers even in the adult animals. Moreover, they encourage researchers to explore the neural functions of TGF-βs present in the adult brain in other regions as well.

## 3. TGF-β in Cerebral Ischemia

The brain is highly sensitive to ischemic insult. Brain ischemia may result from stroke, heart attack, arterial occlusion, and virtually any type of invasive surgery. Ischemic stroke alone is one of the leading causes of death and a leading cause of serious, long-term disability in developed countries. Options for prevention and treatment are limited necessitating new approaches. One of these is TGF-β, a group of endogenous proteins with potential neuroprotective actions.

### 3.1. The Induction of TGF-βs in Response to Brain Ischemia

An increase in the level of TGF-β has been reported in different models of experimental brain ischemia. In twenty-one-day-old rats, a unilateral ligation of the right carotid artery followed by inhalational hypoxia resulted in selective neuronal loss in cortical layer 3 and in the hippocampus of the ligated hemisphere followed by TGF-β1 expression in these sites seventy-two hours after hypoxia [44]. Transient global ischemia also caused an elevation in TGF-β1 levels in the adult hippocampus. Six hours after ischemia, a diffuse expression of TGF-β1 mRNA was found throughout the brain, which further intensified until day 2 and thereafter subsided. In parallel, a massive increase of signal was observed in the hilus of the dentate gyrus and in the CA1 region. Peak levels of TGF-β1 mRNA were found in the hilus around day 4, whereas expression in the CA1 region persisted through day 21 [45]. It is less well known how levels of other subtypes of TGF-βs change after ischemia. A study, which confirmed the induction of TGF-β1 following transient forebrain ischemia actually showed a decrease in the levels of TGF-β2 and -β3 in CA1 region [46] while some other studies reported increases in the levels of TGF-β2 and -β3, their receptors and binding proteins following ischemia [47,48]. The induction of TGF-β1 was also demonstrated following focal ischemic attacks. In rats, as well as in baboons, middle cerebral artery occlusion (MCAO) resulted in a marked expression of TGF-β1 in the penumbral zone that surrounded the tissue destined to infarction [16,49,50]. In fact, an elevated level of TGF-β1 in brain tissue was also found in human following ischemic stroke [51].

### 3.2. Cell Types Expressing TGF-βs Following Brain Ischemia

TGF-β1 upregulation in astrocytes and microglia has been reported to be a predominant response to lesion and during pathology [52–54] that results in the induction of reactive phenotypes [55,56]. Under basal conditions, astrocytes were also shown to express TGF-β1 in some brain regions [38,57]. However, neuronal expression of TGF-β1 has also been reported [52,58,59]. The available data on the cell type specific expression of other TGF-β subtypes is scarce. However, their distributions suggest a dominant neuronal expression [15,16]. Following MCAO, it has been demonstrated in double labeling studies that activated microglia and macrophages are the major source of TGF-β1 mRNA following experimental focal cerebral ischemia [50,60]. However, double-staining experiments demonstrated increased expression of all TGF-β isoforms in astrocytes, too, suggesting that TGF-βs in astrocytes are also important endogenous mediators in the penumbral response to ischemic injury [46]. Furthermore, a rapid up-regulation and persistent expression of TGF-β1 was found in surviving CA1 pyramidal cells after cerebral ischemia supporting that neurons may also contribute to the elevated TGF-β1 levels after ischemia [48].

### 3.3. Effect of TGF-βs on Ischemic Brain Lesion

A neuroprotective role of the induced TGF-β1 following ischemia was suggested by its correlation with the reduction of the infarct area. Thus, clenbuterol, a β(2)-adrenoceptor agonist, was neuroprotective in the hippocampus and caused an increase in TGF-β1 expression in non-ischemic rats and further enhanced TGF-β1 protein levels in rat CA1 pyramidal neurons after transient forebrain ischemia [48]. TGF-β administration itself also decreased the size of infarction. In a rabbit model of thromboembolic stroke, an autologous clot embolus was introduced intracranially. TGF-β1 administered as an intracarotid bolus immediately before autologous clot embolization reduced brain infarct size in a way not related to a direct effect on blood flow [61]. A similar neuroprotective action of exogenously applied TGF-β1 was also found following middle cerebral artery occlusion (MCAO) in the rat [62,63]. Furthermore, the overexpression of TGF-β1 achieved through adenoviral gene transfer also reduced the infarct size in mice following 30 min of MCAO and 1 to 7 days of reperfusion [64].

More relevantly for potential clinical applications, intranasal administration of TGF-β as a noninvasive method for delivery of neuropeptides into the brain to bypass the BBB, reduced infarct volume and improved functional recovery in mice after MCAO [65]. A more direct evidence for the involvement of TGF-β in endogenous neuroprotection was suggested by a study antagonizing TGF-β actions. Injection of a soluble TGF-β type II receptor as a TGF-β antagonist into the brain aggravated the volume of infarction following a 30-minute reversible cerebral focal ischemia [66]. Based on the available evidence, the involvement of TGF-β1 in neuroprotection against ischemia is likely. Since some of the experiments did not differentiate between the subtypes, a role of TGF-β2 and -β3 is also conceivable. The possible mechanisms include anti-inflammatory actions, promotion of scar formation, anti-apoptotic actions, protection against excitotoxicity, and the promotion of angiogenesis and neuroregeneration. These mechanisms will be discussed in Chapter 8 entitled: The neuroprotective mechanisms of TGF-βs. In addition, although the evidence is scarce, TGF-βs, as endogenous neuroprotective proteins, could participate in ischemic tolerance or preconditioning, too [67,68]. The mechanisms of this potential action of TGF-βs might include complex but as yet largely unexplored actions on the gene expression patterns of different neuronal cell types.

## 4. TGF-β in Traumatic CNS Injury

Brain and spinal cord injury continues to result in high morbidity and mortality throughout the world. The neural damage following traumatic injury is a result of direct or primary injury and delayed indirect or secondary mechanisms. An effective neuroprotective agent is still not available to counteract secondary damage caused by traumatic injury. TGF-β is subject of strategies to develop drugs that are able to reduce the indirect tissue damage [69].

### 4.1. The Induction of TGF-βs in Response to Traumatic Injury

An increased level of TGF-βs was found in the brain following traumatic injuries [69]. No clear arterio-jugular venous gradients were apparent providing evidence for the cerebral production of TGF-βs [70]. Indeed, the mRNA encoding TGF-β1 increased in rat cerebral cortex after a penetrating brain injury. A strong expression of TGF-β1 was found 4 days after the lesion in cells within and in the vicinity of the wound [71]. Staining of adjacent sections with an antibody specific for macrophages and microglia/brain macrophages revealed a similar pattern of positive cells, suggesting that invading macrophages, and perhaps reactive microglia, are the source of TGF-β1 in injured brain after a penetrating injury [71]. TGF-β expression was also investigated in a model of spinal cord injury in rats using an impactor. TGF-β expression increased immediately after spinal cord injury in the injured segment and persisted for 24 h after injury [72]. In addition to TGF-β1, TGF-β2 might also be induced by trauma: in a model of fluid-percussion injury, biphasic production of TGF-β2 was detected in the ipsilateral cortex using a bioassay, with a first peak at 30 min and a second peak at 48 h after the lesion [73].

Apart from animal models, the spatial and temporal expression patterns of TGF-β1 and TGF-β2 were also investigated in the human spinal cord after traumatic injury using immunohistochemistry. In control cases, TGF-β1 was confined to occasional blood vessels, intravascular monocytes and some motoneurons, whereas TGF-β2 was only found in intravascular monocytes. After traumatic spinal cord injury, TGF-β1 immunoreactivity was dramatically upregulated by 2 days after injury and was detected within neurons, astrocytes and invading macrophages. The staining was most intense over the first weeks after injury but gradually declined by 1 year. TGF-β2 immunoreactivity was first detected 24 days after injury. It was located in macrophages and astrocytes and remained elevated for up to 1 year [74].

The expression of receptors to TGF-βs was examined by *in situ* hybridization histochemistry in transcortical knife lesions of the striatum in the mouse brain. Type I and type II TGF-β receptor mRNAs were barely detected in the intact brain and first found in meningeal cells near the lesion 1 day postinjury. Many cells expressing TGF-β receptors were found around the lesion site 3 days postinjury, and some of them were immunoreactive for fibronectin. After 5 days postinjury, many fibroblasts migrated from the meninges to the lesion site formed the fibrotic scar, and most of them expressed TGF-β receptors. In contrast, few of reactive astrocytes expressed the receptors throughout the postinjury period examined indicating that meningeal fibroblasts not reactive astrocytes are a major target of TGF-β1 that is upregulated after CNS injury [17].

### 4.2. TGF-β Actions for Traumatic Injury

Transgenic mice overexpressing TGF-β1 in astrocytes developed a severe hydrocephalus, seizures, and early runting [75]. While unmanipulated heterozygous transgenic mice from a low expressor line showed no such alterations, increasing TGF-β1 expression in this line by injury-induced astroglial activation or generation of homozygous offspring did result in the abnormal phenotype. Overexpression of TGF-β1 gene by adenoviral delivery into guinea pig cochleae 4 days prior to injecting an ototoxic dose of aminoglycosides resulted in better hearing and fewer missing inner hair cells. Cochleae with TGF-β1 overexpression exhibited fibrosis in the scala tympani suggesting that the adenovirus-mediated overexpression of TGF-β1 can be used to protect cochlear hair cells and hearing from ototoxic trauma [76].

The area of secondary damage around a traumatic injury has similarity to the ischemic penumbra suggesting a neuroprotective action of TGF-βs in this area. While some evidence is indeed available for such a role of TGF-βs (discussed above), further work is required for establishing and utilizing this function of TGF-βs for traumatic brain injuries.

## 5. TGF-β in Multiple Sclerosis

Multiple sclerosis (MS) is characterized by multiple symptoms of brain and spinal cord dysfunction that reflect degeneration of particular areas of the nervous system that are involved. The affected regions vary between patients and are not specific to the disease. The pathological hallmark is inflammatory demyelination and axonal lesions. Inflammation is primarily driven by autoreactive lymphocytes, which recruit immune cells, such as macrophages, causing tissue damage [77–79].

Experimental autoimmune encephalomyelitis (EAE) is the most widely accepted animal model of MS. Different types of EAE have been developed in order to investigate pathogenetic, clinical and therapeutic aspects of the heterogenic human disease [80]. EAE is characterized by the activation of immune cells, demyelination of axons in the CNS, and paralysis.

### 5.1. The Expression Level of TGF-βs in EAE and MS

TGF-β1 synthesis in glial cells and TGF-β induced signaling in the CNS were activated several days before the onset of paralysis in mice with autoimmune encephalomyelitis. While early production of TGF-β1 was observed in glial cells TGF-β signaling was activated in neurons and later in infiltrating T cells in inflammatory lesions [81]. In actively induced EAE, *in situ* hybridization revealed strong expression of TGF-β1 in meningeal and perivascular mononuclear infiltrates at onset of the disease, continued expression in perivascular infiltrates and scattered mononuclear cells at maximal disease severity, and expression in scattered parenchymal cells during recovery [82]. Double labeling studies revealed subpopulations of infiltrating T-cells to be the major source of TGF-β1 early in the disease, followed by macrophages at peak severity and microglial cells during the recovery phase EAE. Astrocytes and neurons did not express TGF-β1. TGF-β1 expressed early in the disease by T-cells may contribute to inflammatory lesion development, microglial cells may potentially contribute to recovery by expressing immunosuppressive TGF-β1 during remission [82].

Apart from experimental animal models, TGF-β1 was also found to be elevated in MS patients. Using an anchorage-independent growth assay for TGF-β, an increased activity was detected in the supernatants from blood cell cultures from patients with MS in an early study [83]. MS was also associated with increased TGF-β mRNA expressing cells in blood [84,85]. However, more recent studies found a reduced level of TGF-β1 in the serum of MS patients [86]. Furthermore, in a pathway-focused expression profiling of the peripheral blood, reduction in the levels of TGF-β regulated genes was found indicating an overall reduction in TGF-β signaling in MS [87]. Overall, the analysis of TGF-β in blood resulted in controversial data. Furthermore, the examination of cerebrospinal fluid is more valuable in the context of MS. First, routine diagnostic evaluation of cerebrospinal fluid cell counts and various forms of immunoglobulin determination are important to differentiate MS from other diseases. Second, because MS is an organ-specific inflammatory disease and cerebrospinal fluid is often the closest one can get to the target organ [88]. TGF-β levels in cerebrospinal fluid of MS patients were significantly higher in remission than in the active phase [89]. Biologically active TGF-β1 in cerebrospinal fluid correlated positively with the duration of the acute relapse in patients with primary-relapsing MS. The more relapses the patients had the higher the biologically active TGF-β1 was in cerebrospinal fluid [86]. In perivascular and parenchymal macrophages and in hypertrophic astrocytes a strong to intense immunoreactivity was apparent for all 3 TGF-β isoforms and their receptors in active demyelinating MS lesions [90].

### 5.2. The Effects of TGF-βs in EAE and MS

TGF-β1, injected daily for 1–2 weeks, protected against relapsing experimental allergic encephalomyelitis, the animal model of MS. When administered during induction of the disease, TGF-β1 delayed the onset of relapsing experimental allergic encephalomyelitis. However, when administered during a remission, TGF-β1 prevented the occurrence of relapses in relapsing experimental allergic encephalomyelitis [91]. This effect of TGF-β1 may be based on its ability to selectively suppress autoantigen-induced upregulation of pro-inflammatory cytokines [92]. Alternatively, TGF-β1 acts on oligodendrocyte development by modulating the growth factor repertoire in MS to promote remyelination [93]. Nasal administration of TGF-β1 to EAE mice improved clinical EAE. The development and relapse of protracted-relapsing EAE in rats were inhibited [94]. Later, it has been suggested that TGF-β1-induced suppression of EAE is associated with apoptosis of CD4^+^ T cells [95]. However, TGF-β1 may also have actions that are not beneficial in MS. TGF-β enhanced the capability of myelin basic protein-specific Lewis rat T cell lines to transfer EAE [96]. TGF-β1 also re-induced Jagged1 in astrocytes and thereby inhibited process outgrowth from primary human oligodendrocytes [97]. Jagged I is a ligand of the Notch1 receptor whose signaling regulates oligodendrocyte progenitor differentiation and myelin formation in development, and during remyelination in the adult CNS [98]. Furthermore, systemic treatment with a pharmacological inhibitor of TGF-β signaling ameliorated the paralytic disease [81]. Thus, early production of TGF-β1 in the CNS may create a permissive and dangerous environment for the initiation of autoimmune inflammation.

### 5.3. Genetic Studies on the Involvement of TGF-βs in MS

Genome screenings in multiple sclerosis (MS) have identified multiple susceptibility regions supporting a polygenic model for this disease. Evidence for linkage was observed at chromosome 19q13 suggesting the presence of an MS gene(s) in this region encoding TGF-β1. A comprehensive evaluation of common polymorphisms within the TGF-β1 was performed in MS families. Distinct clinical phenotypes were also examined and an association between a TGF-β1 haplotype and a mild disease course was present raising the possibility that TGF-β1 may influence disease expression [99]. However, another study did not find the genetic variation in TGF-β1 as a major factor to susceptibility to MS [100]. In turn, a recent study identified a TGF-β1 genotype with higher frequency in patients suggesting that this polymorphism may play a role in susceptibility to MS [101].

### 5.4. TGF-β Subtypes in MS

The potential role of TGF-β subtypes other than TGF-β1 in MS received much less attention. The available data suggest that acute active lesions exhibit selective TGF-β2 immunoreactivity in ramified microglia encircling the lesion. In contrast, astrocytes within chronic active white matter lesions expressed all three subtypes. Chronic active lesions extended into cortex exhibited selective cortical astrocyte TGF-β2 expression, which suggests that TGF-β cytokines are locally expressed in demyelination and that TGF-β2 may be uniquely regulated [102]. However, an indirect association analysis of TGF-β2 using 8 haplotype-tagging SNPs in a population of 937 MS patients and 2022 controls did not find evidence for association with susceptibility or progression of MS [103]. Thus, it seems likely that TGF-β1 is the major TGF-β subtype involved in MS and other subtypes may play only minor roles in the disease.

## 6. TGF-β in Neurodegenerative Disorders

Neurodegenerative diseases include a group of illnesses that are characterized by a progressive loss of neuronal populations in the CNS. On the basis of the distribution of the degenerating neurons, the illnesses can be classified as diffuse or generalized atrophy, such as Alzheimer’s disease, or a system-specific atrophy, e.g., Parkinson’s and Huntington’s disease. The pathogenesis of neurodegenerative cell losses is largely unknown [104]. Substantial evidence suggests the involvement of TGF-βs in Alzheimer’s and Parkinson’s diseases, which will be the subject of this chapter.

### 6.1. Alzheimer’s Disease

Alzheimer’s disease is a progressive, degenerative disease of the brain that is the most common cause of dementia in the elderly. Typical pathological features of Alzheimer’s disease (AD) are neuritic plaques and neurofibrillary tangles occurring primarily in the cholinergic basal forebrain and the hippocampus, frontal, parietal and temporal lobes of the cerebral cortex. AD is characterized by the presence of beta-amyloid (Aβ) plaques, neurofibrillary tangles, and neuronal loss. Current drugs for AD are only symptomatic, but do not interfere with the underlying pathogenic mechanisms of the disease. The identification of the molecular determinants underlying AD pathogenesis is a fundamental step to design new disease-modifying drugs [105]. An increasing amount of evidence suggests a neuroprotective role for TGF-β1 against Aβ toxicity both *in vitro* and *in vivo* models of AD [106].

#### 6.1.1. TGF-β Levels in AD and Its Animal Models

In mouse models of AD, the expression level of TGF-β1 is increased [107]. In the brains of patients with AD, an increased expression of TGF-β2 was demonstrated [108]. Furthermore, a significant increase in TGF-β1 mRNA expression level in AD patients was found before the manifestation of overt dementia suggesting that it is mobilized in the developmental course of AD pathology [109]. TGF-β1 expression levels correlate with the degree of cerebrovascular amyloid deposition in AD and TGF-β1 immunoreactivity in such cases is increased along the cerebral blood vessels [110]. Although the mechanisms of induction of TGF-β1 are not fully established, acidosis might play a role. A fourfold TGF-β1 bioactivity with decreased intracellular TGF-β1 precursor (latency associated protein-TGF-β1) was found in primary hippocampal cell cultures kept at a mildly acidic pH characteristic of the chronic inflammation accompanying AD [111]. In contrast to the increase in the levels of TGF-βs, the levels of TGF-β type II receptor is reduced in neurons of human AD brain in correlation with pathological hallmarks of the disease [112]. This finding suggests that the overproduction of TGF-β could also be a compensatory mechanism in AD.

#### 6.1.2. The Role of TGF-βs in AD Progression

The attenuation of Aβ neurotoxicity at a pH mildly acidic to the physiological pH was shown to be mediated by the increased activation and signaling of TGF-β [111]. Another potential neuroprotective action of TGF-βs is their effect on the invasion of immune cells from the circulation. While local recruitment of brain microglia to sites of amyloid deposition fail at restricting β-amyloid plaque formation, it is becoming increasingly clear that professional phagocytes from the periphery possess Aβ clearance aptitude. TGF-β Smad 2/3 signaling impacts blood-to-brain trafficking of these cells in transgenic mouse models of AD [113]. AD mice that are deficient in peripheral mononuclear phagocyte TGF-β signaling have increased microglia accumulation around β-amyloid plaques and reduced AD-like pathology [114]. Mice overexpressing TGF-β1 lead to a reduction in macrophage activity and migration ability. Furthermore, pre-incubation of endothelial cells with TGF-β1 reduced the ability of endothelial cells to stimulate macrophage activity [115]. Thus, regulating microglia recruitment into the brain is a potential therapeutic strategy to delay or stop the progression of AD.

Chronic overproduction of TGF-β1 triggers a pathogenic cascade leading to AD-like cerebrovascular amyloidosis, microvascular degeneration, and local alterations in brain metabolic activity. AD is frequently associated with these alterations suggesting that TGF-β1 contributes to the development of AD [116]. However, in aged transgenic mice featuring astrocytic TGF-β1 overexpression, neuronal and cognitive indices—the stimulus-evoked neurometabolic response, cortical cholinergic innervation, and spatial memory in the Morris water maze—were intact suggesting that impaired brain hemodynamics and cerebrovascular function are not accompanied by memory impairment in this model [117]. In turn in mice overproducing both Aβ and TGF-β1 peptides, an early progressive decline in cerebrovascular dilatory ability, and a reduction in constitutive nitric oxide synthesis was found. These mice featured deficient neurovascular and neurometabolic coupling to whisker stimulation, cholinergic denervation, cerebral and cerebrovascular Aβ deposition, astrocyte activation, and impaired Morris water maze performance, which gained severity with age [118]. These data suggest that under some circumstances, TGF-β1 could be a factor that contributes to the progress of AD rather than protects against it. TGF-β1 overexpressing mice were also used to test potential treatments for AD. A nasally administered proteosome-based adjuvant activating macrophages and decreasing vascular amyloid formation was administered on a weekly basis for 3 months beginning at age 13 months in TGF-β1 overexpressing mice. Using magnetic resonance imaging (MRI), control animals were demonstrated to have a significant cerebrovascular pathology. In turn, proteosome-based adjuvant prevented further brain damage and pathological changes in the blood-brain barrier and a significant improvement in cognition [110]. It is possible, that the chronic overexpression of TGF-β results in physiological artifacts leading to contradictory data on the neuroprotective role of TGF-β1 in AD as a modest increase in astroglial TGF-β1 production in aged transgenic mice expressing the human beta-amyloid precursor protein resulted in a reduction in the number of parenchymal amyloid plaques in the hippocampus and cerebral cortex, and decreased the number of dystrophic neurites [119].

Apart from the evidence listed above, genetic studies also support the involvement of TGF-β1 in AD pathogenesis as a specific genotype of the TGF-β1 gene increased the risk to develop the depressive symptoms associated with AD [120]. So far, the role of TGF-β1 was almost exclusively studied in AD while the involvement of other types of TGF-βs cannot be excluded and their examination should be included in future studies. Apolipoprotein E4, the most prevalent genetic risk factor of Alzheimer's disease, reduced the levels of TGF-β1, -β2, as well as -β3 in the septum and that of TGF-β3 in the hippocampus suggesting the possible role of different TGF-βs in mediating the pathological effects of apolipoprotein E4 [121].

#### 6.1.3. TGF-β Signaling in AD

Deficiency of TGF-β signaling seems to be an early event in AD pathogenesis [106]. An elevated level of phosphorylated Smad2 was found in hippocampal neurons of AD brains. However, in contrast to an expected nuclear localization, phosphorylated Smad2 in Alzheimer’s disease was predominantly, and ectopically, found in the neuronal cytoplasm, specifically colocalized with neurofibrillary tangles and granulovacuolar degeneration [122,123]. In cultured cells, reduced TGF-β signaling caused neuronal degeneration and resulted in increased secretion of Aβ [112]. Reduced TGF-β1 signaling seems to contribute both to microglial activation and to ectopic cell-cycle re-activation in neurons, two events that contribute to neurodegeneration in the AD brain. Even the direct binding of Smad proteins to the CDK4 promoter inducing transcriptional inhibition of cell cycle-dependent kinase 4 has recently been identified [124]. The neuroprotective features of TGF-β1 indicate the advantage of rescuing TGF-β1 signaling as a means to slow down the neurodegenerative process in AD [125].

### 6.2. Parkinson’s Disease

The initiation of movement is governed by the interaction of the motor cortex, the thalamus, and a circuit consisting of several members of the basal ganglia, including the striatum, globus pallidus and substantia nigra. The underlying pathology for Parkinson’s disease (PD) is the loss of nigrostriatal dopaminergic cells projecting to the striatum [126]. There is ample evidence that TGF-βs promotes the survival of dopaminergic neurons in the substantia nigra.

#### 6.2.1. TGF-β Levels and Their Alterations in PD

TGF-β2 and -β3 could be detected in freshly isolated and cultured mesencephalic cells [127]. Furthermore, lesioning of the mouse nigrostriatal system with 1-metil-4-fenil-1,2,3,6-tetrahidropiridin (MPTP) significantly upregulated TGF-β2 mRNA levels in the striatum [128]. These data suggest that endogenous TGF-βs are available to affect the survival of dopaminergic neurons in the substantia nigra.

#### 6.2.2. The Effect of TGF-β on the Survival of Dopaminergic Neurons in the Substantia Nigra

TGF-βs were shown to promote the *in vitro* survival of tyrosine hydroxylase (TH)-immunoreactive dopaminergic neurons isolated from the embryonic rat mesencephalon floor. TGF-βs also protected dopaminergic neurons against *N*-methyl-4-phenylpyridinium ion toxicity [127]. It has also been demonstrated that the effects of TGF-βs on dopaminergic cells was neither mediated by astroglial cells nor accompanied by an increase in cell proliferation [129,130]. Treatment of cells dissociated from midbrain floor with TGF-β significantly increased the number of TH-positive dopaminergic neurons within 24 h. Neutralization of TGF-β *in vitro* completely abolished the induction of dopaminergic neurons [131].

Neutralizing endogenous Sonic hedgehog protein abolishes the capacity of TGF-β to induce dopaminergic neurons *in vitro*. In turn, Sonic hedgehog cannot induce TH-positive neurons in the absence of TGF-β suggesting an interaction between these factors in the development of TH neurons [131]. The interaction of TGF-βs with other factors regarding the development and survival of mesencephalic dopaminergic cells has also been extensively investigated. Co-localization of the receptors for TGF-β and glial cell line-derived neurotrophic factor (GDNF) was found. Moreover, the relevance of the TGF-β/GDNF synergism is highlighted by the co-storage of TGF-β and GDNF in secretory vesicles of a model neuron, the chromaffin cell, and their activity-dependent release [132]. GDNF was a potent survival factor for these dopaminergic neurons in culture [133]. Application of antibodies neutralizing TGF-β isoforms abolished the neurotrophic effect of GDNF suggesting that TGF-β may be essential for permitting exogenous GDNF to act as a neuroprotective factor [128]. In turn, GDNF rescued the TGF-β neutralization-dependent loss of the TH-positive cells after 8 days *in vitro* suggesting that TGF-β is required for the induction of dopaminergic neurons, whereas GDNF is required for regulating and/or maintaining a differentiated neuronal phenotype [134]. TGF-β also interacts with neurturin and persephin (PSPN), which are also capable of transient induction of dopaminergic neurons [134]. *In vitro*, combined TGF-β/PSPN treatment achieved a yield of approximately 20% TH-positive cells that were less vulnerable against 1-methyl-4-phenyl pyridinium ion toxicity suggesting that the combination of TGF-β with PSPN is a potent inductive cocktail for the generation of dopaminergic neurons [135]. It has also been demonstrated that the transcriptional cofactor homeodomain interacting protein kinase 2 (HIPK2) is required for the TGF-β mediated survival of mouse dopaminergic neurons. The targeted deletion of Hipk2 has no deleterious effect on the neurogenesis of dopaminergic neurons, but leads to a selective loss of these neurons that is due to increased apoptosis during programmed cell death. The function of HIPK2 depends on its interaction with receptor-regulated Smads to activate TGF-β target genes. Dopaminergic neurons from Hipk2(−/−) mutants fail to survive in the presence of TGF-β3. Furthermore, TgF-β3(−/−) mutants show dopaminergic neuron abnormalities similar to those seen in Hipk2(−/−) mutants [136]. The neuroprotective function of TGF-β2 has also been shown against the neurotoxin 6-hydroxydopamine, which induces a Parkinson-like neurodegeneration. A microglial conditioned medium nearly completely protected cerebellar granule neurons challenged with 6-hydroxydopamine. While the fraction of the medium containing molecules <30 kDa completely protected cerebellar granule neurons, fractions containing molecules <10 kDa were not neuroprotective. The exogenous addition of TGF-β2 to the fraction of the medium not containing it (<10 kDa) fully restored the neuroprotective action. Moreover, the neuroprotection was significantly counteracted by an inhibitor of TGF-β2 transduction pathway [137].

*In vivo* evidence also suggests the involvement of TGF-βs in PD. Neutralization of TGF-β *in vivo* at a critical period resulted in a significant reduction in TH-positive neurons in the ventral midbrain floor but not in the locus coeruleus or diencephalon suggesting that TGF-β is required for the induction of mesencephalic dopaminergic neurons. Furthermore, neutralization of TGF-β at a later time period during maturation of mesencephalic dopaminergic neurons when no further inductive cues are required, also resulted in a significant loss of dopaminergic neurons [131]. Interestingly, no apparent phenotype concerning dopaminergic neurons was observed in TGF-β2 single mutant [138] and Tgf-β2(−/−)/gdnf(−/−) double mutant mice [134]. In turn, in adult mice deficient in Smad3, a molecule involved in the intracellular TGF-β1 signaling cascade, dopaminergic neuronal degeneration was found in the rostral portion of the substantia nigra [139].

Neural transplantation is developing into a therapeutic alternative in Parkinson’s disease. A major limiting factor is that only 3–20% of grafted dopamine neurons survive the procedure. Different approaches may be able to reduce cell death and increase survival of grafted neurons. TGF-βs are among other growth factors that may provide hope for improved survival of transplanted neurons in patients with Parkinson's disease, reducing the need for human embryonic donor tissue and increasing the likelihood of a successful outcome [140].

## 7. TGF-β in Neurodegeneration Resulted from Brain Infections

A variety of brain infections lead to lesions of neuronal tissue. These lesions represent diverse mechanisms as pathogens operate with individual strategies. Encephalitis is most frequently caused by viruses but could also be elicited by bacteria. Rabies, polio, and herpes viruses infect neurons, however, some other viruses attack primarily glial cell types [104]. In some infections, TGF-βs protect against collateral damages caused by the immune system, however, they may promote immune evasion and chronic infections in other cases [141].

Borna disease is a virus-induced, immune-mediated encephalomyelitis based on a delayed-type hypersensitivity reaction. Infection with borna disease virus increased the expression of TGF-β in the brain of rats. An up-regulation of TGF-β expression was found in the brain, while the expressions of signal receptors for TGF-β1 were also increased suggesting that members of the TGF-β family are involved in neuronal disorders induced by borna disease virus infection [142]. The severity of clinical symptoms after intracerebral infection of rats with Borna disease virus was reduced after treatment with TGF-β as continuous intraperitoneal injection. The inhibition of the disease was paralleled by a significant reduction of the inflammatory reaction in the brain [143].

For other viruses, TGF-βs could actually contribute to the infections. Rabies virus causes a non-lytic infection of neurons leading to a fatal myeloencephalitis in mammals including humans. Rabies virus avoids induced neuron cell death. In contrast, T cells that migrate into the infected nervous system are killed by apoptosis and inflammation of the infected nervous system is limited [144]. The examination of brain tissue from patients with cytomegalovirus encephalitis showed co-localization of cytomegalovirus inclusions and TGF-β in cells that contained astrocyte-specific glial filaments. In an *ex vivo* murine model of cytomegalovirus-infected astrocytes, the inoculated cells increased the amounts of infectious cytomegalovirus in parallel with increasing levels of TGF-β mRNA and peptide. Astrocyte release of cytomegalovirus declined in the presence of antibody to TGF-β and increased substantially after the addition of exogenous TGF-β suggesting that cytomegalovirus infection of astrocytes induces the production of TGF-β, which in turn enhances productive cytomegalovirus expression [145]. Progressive multifocal leukoencephalopathy is a fatal demyelinating disease of the CNS. JC virus (JCV), a human neurotropic polyomavirus, is the etiologic infectious agent of this disease. High levels of TGF-β1 and Smads 3 and 4 were detected in JCV-infected oligodendrocytes, which augment the transcription of the JCV promoter in glial cells [146].

Infection by human immunodeficiency virus type 1 (HIV-1) is often complicated with a high incidence of neurologic disorders. By attacking immune cells, HIV-1 infections have special usage of TGF-βs. It is believed that HIV-1, in addition to infecting both macrophage and microglial cells, may also influence the expression of several strategic genes of uninfected neighboring or latently infected brain cells. The HIV-1 Tat protein was demonstrated to increase the expression of TGF-β1 and possibly also its receptor [147–149]. In the cerebrospinal fluid of patients with HIV-1 infection, TGF-β1 level was also significantly elevated [150]. Tat is a transcription transactivator produced by HIV-1 at the early phase of infection and plays a critical role in the expression and replication of the viral genome. This 86 amino acid protein, which can be secreted from the infected cells, has the ability to enter uninfected cells and exert its activity upon the responsive genes. In addition to the HIV-1 promoter, Tat has the capacity to induce transcription of a variety of cellular genes including TGF-βs [148]. In particular, the expression of TGF-β1 in ameboid microglia may play a role in HIV-1 neuropathogenesis [151]. In turn, TGF-β1 may control HIV-1 expression in the brain and astrocytosis, the two hallmarks of brain in AIDS patients [152]. In later stages of HIV-1 infection as microglial cells deteriorate, HIV-RNA is in inverse proportion to TGF-β1. Furthermore, in patients with severe AIDS-dementia, TGF-β1 is undetectable [153].

Bacterial infections may also lead to increased TGF-β levels. In the cerebrospinal fluid of patients with bacterial meningitis, a high level of TGF-β1 mRNA was detectable in the cell populations [154]. Although peripheral infections may also lead to increased TGF-β levels in the brain e.g., associated with fever [155], its brain origin has also been proved: injection of bacterial lipopolysaccharide in the right dorsal hippocampus to mimic cerebral bacterial infection resulted in an increase in TGF-β1 expression 24 h after the injection [67]. TGF-β was suggested to prevent the microvascular changes associated with brain edema formation in bacterial meningitis, a major intracranial complication leading to brain damage based on its effect to interfere with bacterially induced tumor necrosis factor alpha production, oxygen radical formation and the adhesiveness of granulocytes to endothelial cells [156]. There are, however, some contradictory data available on the role of TGF-β in edema formation. In mice with Streptococcus pneumoniae-induced meningitis, a deletion of TGF-β receptor II on leukocytes was found to prevent meningitis-induced vasculitis [157].

## 8. The Role of TGF-βs in Brain Tumors

High-grade gliomas are the most common primary tumors in the central nervous system (CNS) in adults. Since TGF-β is intimately involved in the regulation of several processes characteristic of human malignant glioma including excessive proliferation, infiltrative growth, angiogenesis and suppression of anti-tumor immune surveillance, TGF-β promises to become a novel target for the experimental therapy of human malignant glioma [158].

### 8.1. Proliferative Actions of TGF-βs in Brain Tumors

TGF-βs possess anti-proliferative control on most cell types including astrocytes. Cultured astrocytes demonstrated a significant decrease in DNA synthesis in response to TGF-β and were arrested in the G(1) phase of the cell cycle with an up-regulation of the cyclin-dependent kinase inhibitor (CdkI) p15 [159]. Furthermore, astrocytes from Smad3 null mice demonstrated a reduced TGF-β-mediated inhibition of growth suggesting the involvement of the Smad signal transduction pathway [159].

High-grade human gliomas secrete TGF-β and can activate latent TGF-β [160]. Yet, they are resistant to its growth inhibitory effects as they develop mechanisms that change the anti-proliferative influence of TGF-β into oncogenic cues. Consequently, TGF-β may be involved in the progression of brain tumors [24]. The dominant hypothesis of TGF-β’s pathogenetic association with malignant transformation has been predicated upon acquisition of resistance to its growth inhibitory effects but the existence of intrinsically opposed regulatory mechanisms influenced by TGF-β are also conceivable [161]. The mechanism of conversion might be explained either by the loss of a putative tumor suppressor gene, which mediates TGF-β’s inhibition of growth or by enhancement of an active oncogenic pathway among hyperdiploid glioblastoma multiforme. The expression of the Smad2 and Smad3 proteins is reduced in many glioma cell lines. The phosphorylation and nuclear translocation of Smad2 and Smad3 are also impaired [162]. The loss of p15(INK4B) may also explain the selective loss of growth inhibition by TGF-β in gliomas to form a more aggressive tumor phenotype [159]. TGF-β also induced expression of Sox2, a stemness gene, and this induction was mediated by Sox4, a direct TGF-β target gene. Inhibitors of TGF-β signaling drastically deprived tumorigenicity of glioblastoma cells identifying the relevance of the TGF-β-Sox4-Sox2 pathway, too [163].

Among TGF-β isoforms, TGF-β2 has been identified as the most important factor in the progression of malignant gliomas. TGF-β2, originally described as “glioblastoma-derived T-cell suppressor factor”, was particularly associated with the immuno-suppressed status of patients with glioblastoma. Furthermore, elevated TGF-β2 levels in tumors and in the plasma of patients have been associated with advanced disease stage and poor prognosis [164].

### 8.2. The TGF-β System in the Treatment of Brain Tumors

A variety of different *in vitro* paradigms and rodent glioma models demonstrated that the antagonism of TGF-β holds promise for the treatment of glioblastoma. In particular antisense strategies, inhibition of pro-TGF-β processing, scavenging TGF-β by decorin, or blocking TGF-β activity by specific TGF-β receptor I kinase antagonists have been tested [165,166]. Among these possibilities, the antisense oligonucleotide trabedersen (AP 12009) that specifically blocks TGF-β2 mRNA has the highest potential at present to treat gliobastomas [164]. In addition, the inhibition of TGF-β may also be used as a supplementary treatment as it has the potential to enhance the therapeutic efficacy of glioma-associated antigen vaccines [167].

## 9. The Neuroprotective Mechanisms of TGF-βs

The mechanisms how TGF-βs exert their neuroprotective functions remain to be elucidated. There are, however, several plausible possibilities based on the reported actions of TGF-βs.

### 9.1. Anti-Inflammatory Action

Lesions in the CNS destroy the blood-brain barrier and provoke the invasion of hematogenous cells into the neural tissue. Invading leukocytes, macrophages and lymphocytes secrete various cytokines that induce an inflammatory reaction in the injured CNS and result in local neural degeneration, formation of a cystic cavity and activation of glial cells around the lesion site [168]. Some T helper 1 cytokines such as interferon-gamma, lymphotoxin and tumor necrosis factor have been implicated in driving the immunopathological processes whereas some T helper 2 cytokines, e.g. TGF-βs and interleukin-10 oppose them [169]. TGF-β protects against collateral damages caused by the immune system, by acting as a potent immune suppressor through inhibition of proliferation, differentiation, activation, and effector function of immune cells. Paradoxically, TGF-β may also display pro-inflammatory properties by promoting immune evasion as potent chemoattractant for neutrophils [141]. Within the brain, microglial cells, the brain homologues of macrophages, are also a major source of these cytokines. Activation of microglia is a hallmark of brain pathology. The inflammatory response is mediated by the activated microglia, the resident immune cells of the CNS, which normally respond to neuronal damage and remove the damaged cells by phagocytosis. However, it remains controversial whether microglial cells have beneficial or detrimental functions in various neuropathological conditions [170]. The chronic activation of microglia may in turn cause neuronal damage through the release of potentially cytotoxic molecules such as pro-inflammatory cytokines, reactive oxygen intermediates, proteinases, and complement proteins [171]. TGF-βs were reported to inhibit microglial cells, and thereby exert an anti-inflammatory action. Since microglial cells are a major source of TGF-β1 in the CNS [172], they might exert an auto-inhibitory control on microglial cells [69].

Pathological examination of TGF-β1 null mice revealed an excessive inflammatory response with massive infiltration of lymphocytes and macrophages in many organs suggesting a prominent role for TGF-β1 in homeostatic regulation of immune cell proliferation and extravasation into tissues [173]. In turn, transgenic mice that overexpressed bioactive TGF-β1 in the CNS were more susceptible to the immune-mediated experimental autoimmune encephalomyelitis. TGF-β1 transgenic mice showed an earlier onset of clinical symptoms, more severe disease, and increased mononuclear cell infiltration demonstrating that local expression of TGF-β1 within the brain parenchyma can enhance immune cell infiltration and intensify the CNS impairment resulting from autoimmune responses [174]. Overexpression of TGF-β1 can downregulate the expression of some inflammatory proteins suggesting that the neuroprotective effect of TGF-β1 may result from the inhibition of chemokines during brain injury [64]. The anti-inflammatory action of TGF-β2 has also been suggested. In degenerating optic nerves, astrocytes strongly and continuously expressed TGF-β2 immunoreactivity. Furthermore, TGF-β2 suppressed spontaneous myelin phagocytosis by microglia/macrophages in a mouse *ex vivo* assay of Wallerian degeneration [175]. The anti-inflammatory actions of TGF-βs may also provide the basis for the effect of TGF-β to prevent the microvascular changes associated with brain edema formation [156].

### 9.2. Scar Formation

A brain injury is followed by a cascade of cellular and molecular mechanisms resulting in a secondary injury. To isolate the damaged tissue scar formation takes place in the injured brain. In the process of the fibrotic scar formation, meningeal fibroblasts invade and proliferate in the lesion site to secrete extracellular matrix proteins, such as collagen and laminin. Thereafter, end feet of reactive astrocytes elaborate a glia limitans surrounding the fibrotic scar [17]. Astrogliosis, whereby astrocytes in the CNS become reactive in response to tissue damage, is a prominent process leading to the formation of the glial scar that inhibits axon regeneration after CNS injury. Upon becoming reactive, astrocytes undergo various molecular and morphological changes including upregulating their expression of GFAP and chondroitin sulfate proteoglycans as well as other molecules that are inhibitory to axon growth [176]. Astrolyosis has dual effects on the restoration of neuronal function. Isolating the injured brain tissue may help to prevent the extension of its damaging effects on the healthy tissue. However, gliosis also hinders the reinnervation of the area thereby preventing regaining its functions. TGF-β1 has been implicated in the formation of the fibrotic scar and glia limitans. Many cells expressing TGF-β receptors were found around the lesion site 3 days postinjury, and some of them were immunoreactive for fibronectin [17]. TGF-βs also promote astrogliosis by contributing to the activation of astrocytes around a lesion. The growth of cultured brainstem astrocytes was significantly enhanced by exposure to TGF-β1 [177]. In a three-dimensional *in vitro* model of neurons and astrocytes within a bioactive matrix, TGF-β1 induced robust astrocyte hypertrophy and increased expression of glial fibrillary acidic protein and chondroitin sulfate proteoglycans [178]. Moreover, local injection of TGF-β antagonists into cerebral wounds reduced glial scarring [179–181]. The cellular localization and temporal pattern of expression of TGF-βs after spinal cord injury suggested that TGF-β2 may also participate in scar formation [182].

As far as the mechanism of TGF-βs induction, the blood protein fibrinogen as well as serum albumin were implicated, which leak into the CNS immediately after blood-brain barrier disruption or vascular damage [168,183]. Fibrinogen can activate the latent TGF-β complex and induce phosphorylation of Smad2 in astrocytes. Depletion of fibrinogen in mice reduces active TGF-β, Smad2 phosphorylation, and glial cell activation brain injury. Furthermore, stereotactic injection of fibrinogen into the mouse cortex is sufficient to induce astrogliosis [184]. In addition to vascular injury, TGF-β1 may also play a role in gliosis in degenerative diseases, which include extensive activation of astrocytes. TGF-β1 secreted by microglial cells and invading macrophages has been correlated with the reactive astrocyte phenotype and glial scar formation in multiple sclerosis [185]. The action of TGF-β on astrogliosis may be mediated by additional factors including connective tissue growth factor [186], TGF-β-inducible gene-h3, a protein secreted from reactive astrocytes at the site of a stab wound [187], and adenine nucleotide translocator 1, an inner mitochondrial membrane protein involved with energy mobilization during oxidative phosphorylation and upregulated by TGF-β in reactive astrocytes [188].

### 9.3. Anti-Apoptotic Property

Cells undergoing apoptosis exhibit very different morphological characteristics and temporal profiles of change from cells undergoing necrosis. Apoptosis has been identified with the internucleosomal fragmentation of DNA. More importantly, apoptosis has been associated with a process of programmed cell death, in which a genetic program is activated which results in the death of the cell [189]. Apoptosis enables the balance between growth and elimination of cells and occurs physiologically during the embryonic development or involution processes. Furthermore, infectious agents and other cell-damaging circumstances can lead to apoptosis. Neurons in the penumbra that regained their neuronal functions following ischemia may demonstrate a delayed death by apoptotic processes. Similarly, neurons in tissue undergoing secondary damage around a traumatic lesion may also dye by apoptosis. An increased rate of cell death in the adult nervous system underlies neurodegenerative disease and is a hallmark of multiple sclerosis, Parkinson’s and Alzheimer’s disease [190]. Therefore, anti-apoptotic agents were suggested to promote neuronal survival in a variety of pathological conditions [191].

TGF-βs were shown to inhibit apoptosis. TGF-β1 has also been characterized as an anti-apoptotic factor in a model of staurosporine-induced neuronal death through a mechanism involving activation of the extracellular signal-regulated kinase 1/2 [192] and an inhibition of caspase 3 [193]. Anti-apoptotic action of TGF-β1 on neurons has also been suggested in the dentate gyrus of adult rats following adrenalectomy [194]. The anti-apoptotic property of TGF-βs may contribute to their neuroprotective actions [192]. After adenovirus-mediated TGF-β1 transduction under non-ischemic and ischemic conditions induced by transient middle cerebral artery occlusion, a gradual activation of extracellular signal-regulated kinase 1/2 (Erk1/2) and MAPK-activated protein kinase-1 was found as well as a concomitant increase in Bad phosphorylation in mouse brains [195]. Consistent with these effects, the ischemia-induced increase in Bad protein level and caspase-3 activation was suppressed in TGF-β1-transduced brain. Consequently, DNA fragmentation, ischemic lesions, and neurological deficiency were significantly reduced suggesting that TGF-β1 suppresses Bad expression under lesion conditions, increases Bad phosphorylation, and activates the MAPK/Erk pathway, which may contribute to its neuroprotective activity [195]. Nevertheless, the detrimental effects of homeostasis and the activation of multiple pathways with opposing signals following ischemic stroke indicate that better outcome probably does not depend on a single compound such as TGF-β1 but on several drugs acting in combination at the optimal time in a particular patient [196]. TGF-β pretreatment protected cultured hippocampal neurons from apoptotic degeneration caused by incubation with beta-amyloid peptide (Aβ). Aβ decreased mRNA expression of Bcl-2 expected to promote apoptosis. Incubation with TGF-β before the addition of Aβ reversed these changes even in the presence of Aβ [197]. Furthermore, the neurotoxic effect of Aβ was amplified by SB431542, a selective inhibitor of TGF-β receptor suggesting that TGF-β acts as a factor limiting Aβ toxicity. In pure cultures of rat cortical neurons TGF-β prevented Aβ-induced cell cycle reactivation, whereas lately added TGF-beta1 had no effect on the cell cycle, but rescued the late beta-catenin degradation and tau hyperphosphorylation. The phosphatidylinositol-3-kinase (PI-3-K) inhibitor, LY294402, abrogated all effects suggesting that TGF-β blocks the whole cascade of events leading to Aβ neurotoxicity by activating the PI-3-K pathway [198].

As opposed to the effect of TGF-βs on the survival of neurons, they exert apoptotic effects on several other cell types. Growth factors can define the survival of neural stem cells whose vitality is critical for the growth of the developing brain. TGF-β1 exerted apoptotic activity on cultured stem cells [199], glioma cells [200], oligodendroglial progenitor cells [201], and Schwann cells [33]. Particularly relevant for neuroprotective disorders is the apoptotic activity of TGF-β1 on microglial cells participating in the immune response of the nervous system. In a study testing the effect of TGF-β1 on microglia, astrocytes and oligodendrocytes from newborn rats, TGF-β1 selectively induced apoptosis of microglia, and not of astrocytes or oligodendrocytes. A direct effect of TGF-β1 was implied as the expressions of other cytokines did not change [202]. Furthermore, the relative protein expression of bcl-2 in microglia was not related to frequency of microglial apoptosis suggesting that TGF-β1-induced microglial apoptosis was regulated by a bcl-2-independent mechanism [203].

Among TGF-βs, most experiments either did not discriminate between the subtypes or were specific for TGF-β1. In turn, the role of TGF-β2 and -β3 in mediating apoptosis in non-neuronal tissue was also established. Programmed cell death was significantly reduced in the intestinal mucosa of TGF-β2(+/−) and TGF-β3(+/−) heterozygous mice. This decrease in apoptosis was accompanied by an increase in villus length while proliferation remained unchanged. The level of Bcl-xL and Bcl-2 was significantly up-regulated in TGF-β2(+/−) and TGF-β3(+/−) mice suggesting that TGF-β2 and TGF-β3 play an important role in mediating apoptosis in the intestinal mucosa [204].

### 9.4. Protection against Excitotoxicity

TGF-βs may also affect synaptic functions. Recently, it was demonstrated that TGF-β1 reduced synaptic transfer [35] suggesting that TGF-βs might be able to play a role in counteracting excitotoxic neuronal loss due to the excessive release of glutamate in the peri-infarct area.

TGF-β1 added to cultured cerebral cortical neurons, increased the viability of the cultures determined using trypan blue exclusion. TGF-β1 also significantly reduced the excitotoxic neuronal damage in a concentration-dependent manner suggesting that TGF-β1 has the capacity to diminish the deleterious consequences of an excitotoxic insult [205]. TGF-β1 also protected against chronic glutamate toxicity in organotypic tissue cultures [206]. It was also possible to antagonize the protective effects exerted by TGF-β1 against excitotoxicity. A soluble receptor of TGF-β1 eliminated the neuroprotective activity of TGF-β1 in *N*-methyl-d-aspartate (NMDA)-induced excitotoxic cell death *in vitro* as well as *in vivo* [66]. Later, it has been suggested that PAI-1, an inhibitor of the serine protease tissue-type plasminogen activator might mediate the neuroprotective activity of TGF-β1 against NMDA receptor-mediated excitotoxicity [207]. Some evidence is also available for a contribution of TGF-β1 in excitotoxic damage. Intraneocortical injection of ibotenate, a glutamate analog, in newborn mice produces damage mimicking lesions observed in human infants with cerebral palsy. Pretreatment with TGF-β1 exacerbated the ibotenate-induced lesion while a TGF-β1 neutralizing antibody diminished it [208].

TGF-β subtypes other than TGF-β1 were also implicated in excitotoxicity. TGF-β1 and -β3 but not TGF-β2 were able to prevent the degeneration of cultured chick embryo telencephalic neurons that had been exposed to 1 mM l-glutamate for a period of 60 min [209]. In addition, neuronal death induced by brief NMDA exposure in both mixed neuronal-glial and pure neuronal cultures was increased by TGF-β2 with a similar dose-response curve. These findings indicate that TGF-β2 may potentiate excitotoxicity [210].

Information on the mechanism how TGF-βs affect excitotoxicity is scarce. TGF-β1 augmented the expression of some key subunits of alpha-amino-3-hydroxy-5-methyl-4-isoxazoleproprionic acid (AMPA) and NMDA receptors in the hippocampus. Treatment of cultured hippocampal neurons with TGF-β1 enhanced glutamate-evoked currents [211].

### 9.5. Angiogenesis

TGF-β was shown to promote angiogenesis. When injected subcutaneously in newborn mice, TGF-β induced angiogenesis and activated fibroblasts to produce collagen at the site of injection. In addition, *in vitro* studies showed that TGF-β caused marked increase of proline and leucine incorporation into collagen in rat and human fibroblasts cell lines [212]. A direct effect of TGF-β on angiogenesis was later questioned. It has been suggested that TGF-β1 can potentiate the effect of other growth factors. Indeed, TGF-β1 increased the effect of vascular endothelial growth factor (VEGF)-induced angiogenesis in a model, in which microvascular endothelial cells formed capillary-like tubes within collagen gels [213]. Using a similar three-dimensional hydrated collagen gel as well as two-dimensional Petri dish cultures, low concentrations of TGF-β1 enhanced the stimulatory effects of basic fibroblast growth factor [214]. *In vivo*, in disc angiogenesis system, TGF-β1 also potentiated the proliferation of endothelial cells. To test the role of endogenous TGF-β in the spontaneous angiogenesis of wound healing, a monoclonal anti-TGF-β antibody was applied, which decreased the spontaneous vascular growth below the level of controls [215]. Thus, endogenous TGF-β has the potential to contribute to spontaneous angiogenesis, e.g., during wound healing. In addition, knockout mice for the different components of the TGF-β signaling pathway have shown that TGF-β is indispensable for angiogenesis and mutations in TGF-β receptors have been linked to a vascular disorder named hereditary hemorrhagic telangiectasia [216]. In the brain, similar mechanisms are expected for angiogenesis although the available data is less abundant. A complex interplay between TGF-β1 and vascular endothelial growth factor has been suggested. Both of them induced angiogenesis but had opposing effects on endothelial cells. VEGF protected endothelial cells from apoptosis while TGF-β1 induced apoptosis. Furthermore, the inhibition of VEGF blocked both apoptosis and angiogenesis elicited by TGF-β1 suggesting refined control of angiogenesis by the interplay of these growth factors [217].

Angiogenesis is also an important part of tumor growth *in vivo*. The overproduction of TGF-β1 by tumor cells can contribute to neovascularization and may help promote tumor development *in vivo* [218]. TGF-βs are particularly involved in malignant gliomas characterized by excessive proliferation, infiltrative growth, angiogenesis and suppression of anti-tumor immune surveillance. The effects of TGF-β in glioma biology include a survival advantage for glioma cells by enforced cell growth, migration, invasion, angiogenesis and immune paralysis [158].

### 9.6. Neuronal Regeneration

Establishing a direct role of TGF-β in neuronal regeneration requires an examination of the potential involvement of scar formation because an indirect effect by fibrosis and astrogliosis is always a possibility. For example, continuous infusion of a small molecule inhibitor of type I TGF-β receptor kinase LY-364947 resulted in the regeneration of tyrosine hydroxylase-immunoreactive after a unilateral transection of the nigrostriatal dopaminergic pathway [181]. In the injured brain, infusion of LY-364947 suppressed fibrotic scar formation and decreased the numbers of reactive astrocytes in the lesion site indicating that TGF-β signaling contributes to the fibrotic scar and the blockade of axonal regeneration [181]. Some evidence also exists on the direct role of TGF-β in neuronal regeneration. When axons of cultured rat hippocampal neurons cut by local irradiation of laser beam, the axonal growth was stopped by laser irradiation. Addition of TGF-β remarkably promoted the axonal re-elongation from the injured site in a concentration dependent manner suggesting that TGF-β1 has a capability of promoting axonal regeneration of brain neurons after lesioning [211,219]. Some of these effects may be mediated by other growth factors. Nerve growth factor (NGF), which is known to influence neuronal development, function, and response to injury, is regulated by at least two promoters that govern synthesis of four different transcripts, A through D. TGF-β1 induced NGF mRNA and protein in rat and mouse glia but not in neurons. Furthermore, transcripts A, B and D, but not C, are upregulated by TGF-beta1 in mouse glia, whereas in rat glia, the major responsive transcript is C [220]. Altogether, these results provide some evidence that NGF might mediate the effects of TGF-β1 on neuroregeneration.

Apart from affecting axonal outgrowth, TGF-βs may also participate in other aspect of neuronal regeneration such as synaptogenesis and the regeneration of dendritic spines. It has been demonstrated that TGF-β1 facilitated synaptogenesis in Xenopus nerve-muscle co-cultures [34] and it may also contribute to changing dendritic morphology following brain injury [221].

## 10. Conclusions

The described mechanisms of actions of TGF-β are not equally well established. The anti-inflammatory action of TGF-βs is fully proved. A substantial amount of evidence is available to support the role of TGF-βs in scar formation including astrogliosis. TGF-β is known to affect cell survival, and an anti-apoptotic effect on neurons is supported by a number of experiments. A role of TGF-βs in excitotoxicity has also been intensively investigated. Similarly, the available evidence is strong for a role of TGF-βs to angiogenesis and their contribution to the vascularization of tumors. Finally, neuronal regeneration might also involve TGF-βs.

The contribution of these mechanisms varies depending on the type of brain lesion (Figure 1). The role of neuroinflammation has been recognized in a diverse range of cerebral pathologies, including ischemic and traumatic brain injury. Sclerosis multiplex is an inflammatory disease, in which an anti-inflammatory protein can play obvious roles. Neurodegenerative disorders might also include an inflammatory aspect. In turn, pathogens often develop ways to protect against inflammatory host defense mechanisms. Therefore, TGF-β may not be very effective against infections.

**Figure 2:**
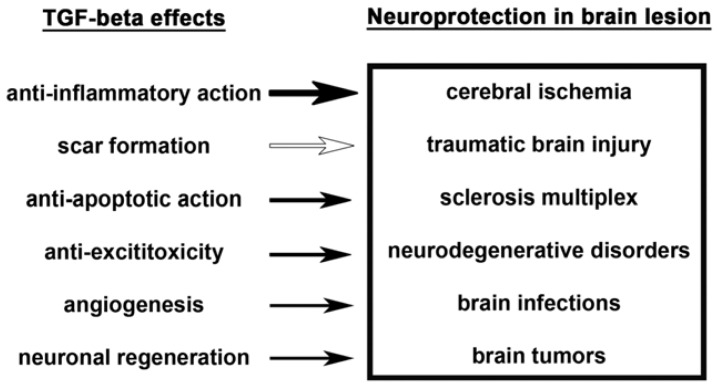
The contribution of TGF-β effects to its neuroprotective actions in different brain lesions. In the left mechanisms are listed, through which TGF-β effects are exerted on neuronal tissue. The size of the arrow indicates the general importance of the particular mechanism in conveying the neuroprotective actions of TGF-β. A white arrow belongs to scar formation because this action of TGF-β may not necessarily be neuroprotective.

Apoptosis is prominent for cerebral ischemia and traumatic brain injury around the area of the primary damage. This is a clinically relevant region because of the realistic chance for preserving this tissue by treatment. Anti-apoptotic and anti-excitotoxic effects of TGF-β could be relevant when aiming at developing neuroprotective agents (Figure 2). Furthermore, the occlusion of cerebral vessels during stroke is followed by proliferation of microvessels, *i.e.*, angiogenesis. This process is also particularly marked in the border zone of the infarct, the ischemic penumbra, where a decreased blood flow is following focal ischemia. This increase in vascularization is another process, in which TGF-β could play a role following brain injuries as increasing blood flow in this vulnerable region can alleviate the consequences of an ischemic attack. The involvement of TGF-β in neuroregeneration is not fully proved while its effect in scar formation remains controversial as to its contribution of neuroprotection following any brain lesion. Therefore, the potential contribution of these mechanisms to the neuroprotective actions of TGF-β is low.

## Figures and Tables

**Table 1:** Brain areas with high expression level of TGF-βs.

Type of TGF-β	Brain Regions
TGF-β1	hippocampus; amygdala, central nucleus; hypothalamus, preoptic and paraventricular nuclei; midbrain; pons and medulla oblongata, reticular formation, motor nuclei, superior olive, area postrema; choroid plexus
TGF-β2	cerebral cortex, layer V; dentate gyrus; thalamus, parafascicular and midline nuclei; hypothalamus, posterior part and mamillary body; midbrain, raphe nuclei; pons and medulla oblongata, reticular formation, motor nuclei, superior olive, area postrema; cerebellar cortex, Purkinje cell layer; choroid plexus
TGF-β3	cerebral cortex, layers IV,VI; dentate gyrus; amygdala, basal nucleus; thalamus, anterior, ventral, midline and reticular nuclei; hypothalamus, arcuate and supramamillary nuclei; midbrain, superior colliculus; pons and medulla oblongata, reticular formation, motor nuclei, superior and inferior olive, area postrema

## References

[1] Roberts A.B., Anzano M.A., Lamb L.C., Smith J.M., Sporn M.B. (1981). New class of transforming growth factors potentiated by epidermal growth factor: Isolation from non-neoplastic tissues. Proc. Natl. Acad. Sci. USA.

[2] Burt D.W., Law A.S. (1994). Evolution of the transforming growth factor-beta superfamily. Prog. Growth Factor Res.

[3] Lawrence D.A. (1996). Transforming growth factor-beta: A general review. Eur. Cytokine Netw.

[4] Clark D.A., Coker R. (1998). Transforming growth factor-beta (TGF-beta). Int. J. Biochem. Cell. Biol.

[5] Funkenstein B., Olekh E., Jakowlew S.B. (2010). Identification of a novel transforming growth factor-beta (TGF-beta6) gene in fish: Regulation in skeletal muscle by nutritional state. BMC Mol. Biol.

[6] Roberts A.B. (1998). Molecular and cell biology of TGF-beta. Miner. Electrolyte. Metab.

[7] Sinha S., Nevett C., Shuttleworth C.A., Kielty C.M. (1998). Cellular and extracellular biology of the latent transforming growth factor-beta binding proteins. Matrix. Biol.

[8] Oklu R., Hesketh R. (2000). The latent transforming growth factor beta binding protein (LTBP) family. Biochem. J.

[9] Khalil N. (1999). TGF-beta: From latent to active. Microbes. Infect.

[10] Konig H.G., Kogel D., Rami A., Prehn J.H. (2005). TGF-β1 activates two distinct type I receptors in neurons: Implications for neuronal NF-κB signaling. J. Cell. Biol.

[11] Gumienny T.L., Padgett R.W. (2002). The other side of TGF-beta superfamily signal regulation: Thinking outside the cell. Trends Endocrinol. Metab.

[12] Ten Dijke P., Hill C.S. (2004). New insights into TGF-beta-Smad signalling. Trends Biochem. Sci.

[13] Schmierer B., Hill C.S. (2007). TGFbeta-SMAD signal transduction: Molecular specificity and functional flexibility. Nat. Rev. Mol. Cell Biol.

[14] Mu Y., Gudey S.K., Landstrom M. (2012). Non-Smad signaling pathways. Cell Tissue Res.

[15] Unsicker K., Flanders K.C., Cissel D.S., Lafyatis R., Sporn M.B. (1991). Transforming growth factor beta isoforms in the adult rat central and peripheral nervous system. Neuroscience.

[16] Vincze C., Pal G., Wappler E.A., Szabo E.R., Nagy Z.G., Lovas G., Dobolyi A. (2010). Distribution of mRNAs encoding transforming growth factors-beta1, -2, and -3 in the intact rat brain and after experimentally induced focal ischemia. J. Comp. Neurol.

[17] Komuta Y., Teng X., Yanagisawa H., Sango K., Kawamura K., Kawano H. (2010). Expression of transforming growth factor-beta receptors in meningeal fibroblasts of the injured mouse brain. Cell. Mol. Neurobiol.

[18] Bottner M., Krieglstein K., Unsicker K. (2000). The transforming growth factor-betas: Structure, signaling, and roles in nervous system development and functions. J. Neurochem.

[19] Dobolyi A., Palkovits M. (2008). Expression of latent transforming growth factor beta binding proteins in the rat brain. J. Comp. Neurol.

[20] Dobolyi A, Contreras C.M. (2012). Transforming Growth Factor Beta in the Central Nervous System. Neuroscience —Dealing with Frontiers.

[21] Bottner M., Unsicker K., Suter-Crazzolara C. (1996). Expression of TGF-beta type II receptor mRNA in the CNS. Neuroreport.

[22] Flanders K.C., Ludecke G., Engels S., Cissel D.S., Roberts A.B., Kondaiah P., Lafyatis R., Sporn M.B., Unsicker K. (1991). Localization and actions of transforming growth factor-beta s in the embryonic nervous system. Development.

[23] Zhang J.M., Hoffmann R., Sieber-Blum M. (1997). Mitogenic and anti-proliferative signals for neural crest cells and the neurogenic action of TGF-beta1. Dev. Dyn.

[24] Aigner L., Bogdahn U. (2008). TGF-beta in neural stem cells and in tumors of the central nervous system. Cell Tissue Res.

[25] Vogel T., Ahrens S., Buttner N., Krieglstein K. (2010). Transforming growth factor beta promotes neuronal cell fate of mouse cortical and hippocampal progenitors *in vitro* and *in vivo*: Identification of Nedd9 as an essential signaling component. Cereb. Cortex.

[26] Mathieu P., Piantanida A.P., Pitossi F. (2011). Chronic expression of transforming growth factor-beta enhances adult neurogenesis. Neuroimmunomodulation.

[27] Battista D., Ferrari C.C., Gage F.H., Pitossi F.J. (2006). Neurogenic niche modulation by activated microglia: Transforming growth factor beta increases neurogenesis in the adult dentate gyrus. Eur. J. Neurosci.

[28] Garcia-Campmany L., Marti E. (2007). The TGFbeta intracellular effector Smad3 regulates neuronal differentiation and cell fate specification in the developing spinal cord. Development.

[29] Gouin A., Bloch-Gallego E., Tanaka H., Rosenthal A., Henderson C.E. (1996). Transforming growth factor-beta 3, glial cell line-derived neurotrophic factor, and fibroblast growth factor-2, act in different manners to promote motoneuron survival *in vitro*. J. Neurosci. Res.

[30] Jiang Y., McLennan I.S., Koishi K., Hendry I.A. (2000). Transforming growth factor-beta 2 is anterogradely and retrogradely transported in motoneurons and up-regulated after nerve injury. Neuroscience.

[31] Jiang Y., Zhang M., Koishi K., McLennan I.S. (2000). TGF-beta 2 attenuates the injury-induced death of mature motoneurons. J. Neurosci. Res.

[32] Guenard V., Rosenbaum T., Gwynn L.A., Doetschman T., Ratner N., Wood P.M. (1995). Effect of transforming growth factor-beta 1 and -beta 2 on Schwann cell proliferation on neurites. Glia.

[33] Parkinson D.B., Dong Z., Bunting H., Whitfield J., Meier C., Marie H., Mirsky R., Jessen K.R. (2001). Transforming growth factor beta (TGFbeta) mediates Schwann cell death *in vitro* and *in vivo*: Examination of c-Jun activation, interactions with survival signals, and the relationship of TGFbeta-mediated death to Schwann cell differentiation. J. Neurosci.

[34] Feng Z., Ko C.P. (2008). Schwann cells promote synaptogenesis at the neuromuscular junction via transforming growth factor-beta1. J. Neurosci.

[35] Heupel K., Sargsyan V., Plomp J.J., Rickmann M., Varoqueaux F., Zhang W., Krieglstein K (2008). Loss of transforming growth factor-beta 2 leads to impairment of central synapse function. Neural. Dev.

[36] Fukushima T., Liu R.Y., Byrne J.H. (2007). Transforming growth factor-beta2 modulates synaptic efficacy and plasticity and induces phosphorylation of CREB in hippocampal neurons. Hippocampus.

[37] Prevot V., Bouret S., Croix D., Takumi T., Jennes L., Mitchell V., Beauvillain J.C. (2000). Evidence that members of the TGFbeta superfamily play a role in regulation of the GnRH neuroendocrine axis: Expression of a type I serine-threonine kinase receptor for TGRbeta and activin in GnRH neurones and hypothalamic areas of the female rat. J. Neuroendocrinol.

[38] Bouret S., de Seranno S., Beauvillain J.C., Prevot V. (2004). Transforming growth factor beta1 may directly influence gonadotropin-releasing hormone gene expression in the rat hypothalamus. Endocrinology.

[39] Dhandapani K.M., Hadman M., de Sevilla L., Wade M.F., Mahesh V.B., Brann D.W. (2003). Astrocyte protection of neurons: Role of transforming growth factor-beta signaling via a c-Jun-AP-1 protective pathway. J. Biol. Chem.

[40] Sortino M.A., Chisari M., Merlo S., Vancheri C., Caruso M., Nicoletti F., Canonico P.L., Copani A. (2004). Glia mediates the neuroprotective action of estradiol on beta-amyloid-induced neuronal death. Endocrinology.

[41] Fevre-Montange M., Dumontel C., Chevallier P., Isnard A.K., Guigard M.P., Trouillas J. (2004). Localization of transforming growth factors, TGFbeta1 and TGFbeta3, in hypothalamic magnocellular neurones and the neurohypophysis. J. Neuroendocrinol.

[42] Beynon A.L., Thome J., Coogan A.N. (2009). Age and time of day influences on the expression of transforming growth factor-beta and phosphorylated SMAD3 in the mouse suprachiasmatic and paraventricular nuclei. Neuroimmunomodulation.

[43] Giacomini D., Paez-Pereda M., Stalla J., Stalla G.K., Arzt E. (2009). Molecular interaction of BMP-4, TGF-beta, and estrogens in lactotrophs: Impact on the PRL promoter. Mol. Endocrinol.

[44] Klempt N.D., Sirimanne E., Gunn A.J., Klempt M., Singh K., Williams C., Gluckman P.D. (1992). Hypoxia-ischemia induces transforming growth factor beta 1 mRNA in the infant rat brain. Brain Res. Mol. Brain Res.

[45] Lehrmann E., Kiefer R., Finsen B., Diemer N.H., Zimmer J., Hartung H.P. (1995). Cytokines in cerebral ischemia: Expression of transforming growth factor beta-1 (TGF-beta 1) mRNA in the postischemic adult rat hippocampus. Exp. Neurol.

[46] Knuckey N.W., Finch P., Palm D.E., Primiano M.J., Johanson C.E., Flanders K.C., Thompson N.L. (1996). Differential neuronal and astrocytic expression of transforming growth factor beta isoforms in rat hippocampus following transient forebrain ischemia. Brain Res. Mol. Brain Res.

[47] Ata K.A., Lennmyr F., Funa K., Olsson Y., Terent A. (1999). Expression of transforming growth factor-beta1, 2, 3 isoforms and type I and II receptors in acute focal cerebral ischemia: An immunohistochemical study in rat after transient and permanent occlusion of middle cerebral artery. Acta Neuropathol.

[48] Zhu Y., Culmsee C., Roth-Eichhorn S., Krieglstein J. (2001). Beta(2)-adrenoceptor stimulation enhances latent transforming growth factor-beta-binding protein-1 and transforming growth factor-beta1 expression in rat hippocampus after transient forebrain ischemia. Neuroscience.

[49] Ali C., Docagne F., Nicole O., Lesne S., Toutain J., Young A., Chazalviel L., Divoux D., Caly M., Cabal P. (2001). Increased expression of transforming growth factor-beta after cerebral ischemia in the baboon: An endogenous marker of neuronal stress?. J. Cereb. Blood Flow Metab.

[50] Doyle K.P., Cekanaviciute E., Mamer L.E., Buckwalter M.S. (2010). TGFbeta signaling in the brain increases with aging and signals to astrocytes and innate immune cells in the weeks after stroke. J. Neuroinflammation.

[51] Krupinski J., Kumar P., Kumar S., Kaluza J. (1996). Increased expression of TGF-beta 1 in brain tissue after ischemic stroke in humans. Stroke.

[52] Wu Z., Hayashi Y., Zhang J., Nakanishi H. (2007). Involvement of prostaglandin E2 released from leptomeningeal cells in increased expression of transforming growth factor-beta in glial cells and cortical neurons during systemic inflammation. J. Neurosci. Res.

[53] Wu Z., Tokuda Y., Zhang X.W., Nakanishi H. (2008). Age-dependent responses of glial cells and leptomeninges during systemic inflammation. Neurobiol. Dis.

[54] Krohn K. (1999). TGF-beta1-dependent differential expression of a rat homolog for latent TGF-beta binding protein in astrocytes and C6 glioma cells. Glia.

[55] Flanders K.C., Ren R.F., Lippa C.F. (1998). Transforming growth factor-betas in neurodegenerative disease. Prog. Neurobiol.

[56] Morgan T.E., Nichols N.R., Pasinetti G.M., Finch C.E. (1993). TGF-beta 1 mRNA increases in macrophage/microglial cells of the hippocampus in response to deafferentation and kainic acid-induced neurodegeneration. Exp. Neurol.

[57] Dhandapani K.M., Brann D.W. (2003). Transforming growth factor-beta: A neuroprotective factor in cerebral ischemia. Cell Biochem. Biophys.

[58] Battaglia G., Cannella M., Riozzi B., Orobello S., Maat-Schieman M.L., Aronica E., Busceti C.L., Ciarmiello A., Alberti S., Amico E. (2011). Early defect of transforming growth factor beta1 formation in Huntington's disease. J. Cell. Mol. Med.

[59] Lacmann A., Hess D., Gohla G., Roussa E., Krieglstein K. (2007). Activity-dependent release of transforming growth factor-beta in a neuronal network *in vitro*. Neuroscience.

[60] Lehrmann E., Kiefer R., Christensen T., Toyka K.V., Zimmer J., Diemer N.H., Hartung H.P., Finsen B. (1998). Microglia and macrophages are major sources of locally produced transforming growth factor-beta1 after transient middle cerebral artery occlusion in rats. Glia.

[61] Gross C.E., Bednar M.M., Howard D.B., Sporn M.B. (1993). Transforming growth factor-beta 1 reduces infarct size after experimental cerebral ischemia in a rabbit model. Stroke.

[62] Henrich-Noack P., Prehn J.H., Krieglstein J. (1994). Neuroprotective effects of TGF-beta 1. J. Neural. Transm. Suppl.

[63] McNeill H., Williams C., Guan J., Dragunow M., Lawlor P., Sirimanne E., Nikolics K., Gluckman P. (1994). Neuronal rescue with transforming growth factor-beta 1 after hypoxic-ischaemic brain injury. Neuroreport.

[64] Pang L., Ye W., Che X.M., Roessler B.J., Betz A.L., Yang G.Y. (2001). Reduction of inflammatory response in the mouse brain with adenoviral-mediated transforming growth factor-ss1 expression. Stroke.

[65] Ma M., Ma Y., Yi X., Guo R., Zhu W., Fan X., Xu G., Frey W.H., Liu X. (2008). Intranasal delivery of transforming growth factor-beta1 in mice after stroke reduces infarct volume and increases neurogenesis in the subventricular zone. BMC Neurosci.

[66] Ruocco A., Nicole O., Docagne F., Ali C., Chazalviel L., Komesli S., Yablonsky F., Roussel S., MacKenzie E.T., Vivien D., Buisson A. (1999). A transforming growth factor-beta antagonist unmasks the neuroprotective role of this endogenous cytokine in excitotoxic and ischemic brain injury. J. Cereb. Blood Flow Metab.

[67] Boche D., Cunningham C., Gauldie J., Perry V.H. (2003). Transforming growth factor-beta 1-mediated neuroprotection against excitotoxic injury *in vivo*. J. Cereb. Blood Flow Metab.

[68] Pera J., Zawadzka M., Kaminska B., Szczudlik A. (2004). Influence of chemical and ischemic preconditioning on cytokine expression after focal brain ischemia. J. Neurosci. Res.

[69] Lenzlinger P.M., Morganti-Kossmann M.C., Laurer H.L., McIntosh T.K. (2001). The duality of the inflammatory response to traumatic brain injury. Mol. Neurobiol.

[70] Helmy A., Carpenter K.L., Menon D.K., Pickard J.D., Hutchinson P.J. (2011). The cytokine response to human traumatic brain injury: Temporal profiles and evidence for cerebral parenchymal production. J. Cereb. Blood Flow Metab.

[71] Lindholm D., Castren E., Kiefer R., Zafra F., Thoenen H. (1992). Transforming growth factor-beta 1 in the rat brain: Increase after injury and inhibition of astrocyte proliferation. J. Cell Biol.

[72] Wang X., Chen W., Liu W., Wu J., Shao Y., Zhang X. (2009). The role of thrombospondin-1 and transforming growth factor-beta after spinal cord injury in the rat. J. Clin. Neurosci.

[73] Rimaniol A.C., Lekieffre D., Serrano A., Masson A., Benavides J., Zavala F. (1995). Biphasic transforming growth factor-beta production flanking the pro-inflammatory cytokine response in cerebral trauma. Neuroreport.

[74] Buss A., Pech K., Kakulas B.A., Martin D., Schoenen J., Noth J., Brook G.A. (2008). TGF-beta 1 and TGF-beta 2 expression after traumatic human spinal cord injury. Spinal Cord.

[75] Wyss-Coray T., Feng L., Masliah E., Ruppe M.D., Lee H.S., Toggas S.M., Rockenstein E.M., Mucke L. (1995). Increased central nervous system production of extracellular matrix components and development of hydrocephalus in transgenic mice overexpressing transforming growth factor-β1. Am. J. Pathol.

[76] Kawamoto K., Yagi M., Stover T., Kanzaki S., Raphael Y. (2003). Hearing and hair cells are protected by adenoviral gene therapy with TGF-beta 1 and GDNF. Mol. Ther.

[77] Hauser S.L., Oksenberg J.R. (2006). The neurobiology of multiple sclerosis: Genes, inflammation, and neurodegeneration. Neuron.

[78] Voumvourakis K.I., Antonelou R., Kitsos D.K., Stamboulis E., Tsiodras S. (2011). TGF-beta/BMPs: Crucial crossroad in neural autoimmune disorders. Neurochem. Int.

[79] Mirshafiey A., Mohsenzadegan M. (2009). TGF-beta as a promising option in the treatment of multiple sclerosis. Neuropharmacology.

[80] Mix E., Meyer-Rienecker H., Hartung H.P., Zettl U.K. (2010). Animal models of multiple sclerosis—Potentials and limitations. Prog. Neurobiol.

[81] Luo J., Ho P.P., Buckwalter M.S., Hsu T., Lee L.Y., Zhang H., Kim D.K., Kim S.J., Gambhir S.S., Steinman L., Wyss-Coray T. (2007). Glia-dependent TGF-beta signaling, acting independently of the TH17 pathway, is critical for initiation of murine autoimmune encephalomyelitis. J. Clin. Invest.

[82] Kiefer R., Schweitzer T., Jung S., Toyka K.V., Hartung H.P. (1998). Sequential expression of transforming growth factor-beta1 by T-cells, macrophages, and microglia in rat spinal cord during autoimmune inflammation. J. Neuropathol. Exp. Neurol.

[83] Beck J., Rondot P., Jullien P., Wietzerbin J., Lawrence D.A. (1991). TGF-beta-like activity produced during regression of exacerbations in multiple sclerosis. Acta Neurol. Scand.

[84] Soderstrom M., Hillert J., Link J., Navikas V., Fredrikson S., Link H. (1995). Expression of IFN-gamma, IL-4, and TGF-beta in multiple sclerosis in relation to HLA-Dw2 phenotype and stage of disease. Mult. Scler.

[85] Link J., Soderstrom M., Olsson T., Hojeberg B., Ljungdahl A., Link H. (1994). Increased transforming growth factor-beta, interleukin-4, and interferon-gamma in multiple sclerosis. Ann. Neurol.

[86] Rollnik J.D., Sindern E., Schweppe C., Malin J.P. (1997). Biologically active TGF-beta 1 is increased in cerebrospinal fluid while it is reduced in serum in multiple sclerosis patients. Acta Neurol. Scand.

[87] Meoli E.M., Oh U., Grant C.W., Jacobson S. (2011). TGF-beta signaling is altered in the peripheral blood of subjects with multiple sclerosis. J. Neuroimmunol.

[88] Olsson T. (1994). Multiple sclerosis:cerebrospinal fluid. Ann. Neurol.

[89] Carrieri P.B., Provitera V., Bruno R., Perrella M., Tartaglia G., Busto A., Perrella O. (1997). Possible role of transforming growth factor-beta in relapsing-remitting multiple sclerosis. Neurol. Res.

[90] De Groot C.J., Montagne L., Barten A.D., Sminia P., van der Valk P. (1999). Expression of transforming growth factor (TGF)-beta1, -beta2, and -beta3 isoforms and TGF-beta type I and type II receptors in multiple sclerosis lesions and human adult astrocyte cultures. J. Neuropathol. Exp. Neurol.

[91] Kuruvilla A.P., Shah R., Hochwald G.M., Liggitt H.D., Palladino M.A., Thorbecke G.J. (1991). Protective effect of transforming growth factor beta 1 on experimental autoimmune diseases in mice. Proc. Natl. Acad. Sci. USA.

[92] Link J., He B., Navikas V., Palasik W., Fredrikson S., Soderstrom M., Link H. (1995). Transforming growth factor-beta 1 suppresses autoantigen-induced expression of pro-inflammatory cytokines but not of interleukin-10 in multiple sclerosis and myasthenia gravis. J. Neuroimmunol.

[93] Diemel L.T., Jackson S.J., Cuzner M.L. (2003). Role for TGF-beta1, FGF-2 and PDGF-AA in a myelination of CNS aggregate cultures enriched with macrophages. J. Neurosci. Res.

[94] Ishikawa M., Jin Y., Guo H., Link H., Xiao B.G. (1999). Nasal administration of transforming growth factor-beta1 induces dendritic cells and inhibits protracted-relapsing experimental allergic encephalomyelitis. Mult. Scler.

[95] Jin Y.X., Xu L.Y., Guo H., Ishikawa M., Link H., Xiao B.G. (2000). TGF-beta1 inhibits protracted-relapsing experimental autoimmune encephalomyelitis by activating dendritic cells. J. Autoimmun.

[96] Weinberg A.D., Whitham R., Swain S.L., Morrison W.J., Wyrick G., Hoy C., Vandenbark A.A., Offner H. (1992). Transforming growth factor-beta enhances the *in vivo* effector function and memory phenotype of antigen-specific T helper cells in experimental autoimmune encephalomyelitis. J. Immunol.

[97] John G.R., Shankar S.L., Shafit-Zagardo B., Massimi A., Lee S.C., Raine C.S., Brosnan C.F. (2002). Multiple sclerosis: Re-expression of a developmental pathway that restricts oligodendrocyte maturation. Nat. Med.

[98] Zhang Y., Zhang J., Navrazhina K., Argaw A.T., Zameer A., Gurfein B.T., Brosnan C.F., John G.R. (2010). TGFbeta1 induces Jagged1 expression in astrocytes via ALK5 and Smad3 and regulates the balance between oligodendrocyte progenitor proliferation and differentiation. Glia.

[99] Green A.J., Barcellos L.F., Rimmler J.B., Garcia M.E., Caillier S., Lincoln R.R., Bucher P., Pericak-Vance M.A., Haines J.L., Hauser S.L., Oksenberg J.R. (2001). Sequence variation in the transforming growth factor-beta1 (TGFB1) gene and multiple sclerosis susceptibility. J. Neuroimmunol.

[100] Weinshenker B.G., Hebrink D., Kantarci O.H., Schaefer-Klein J., Atkinson E., Schaid D., McMurray C.M. (2001). Genetic variation in the transforming growth factor beta1 gene in multiple sclerosis. J. Neuroimmunol.

[101] Izad M., Vodjgani M., Niknam M.H., Amirzargar A., Shahbeigi S., Heidari A.B., Keramatipour M. (2010). Cytokines genes polymorphisms and risk of multiple sclerosis. Am. J. Med. Sci.

[102] Peress N.S., Perillo E., Seidman R.J. (1996). Glial transforming growth factor (TGF)-beta isotypes in multiple sclerosis: Differential glial expression of TGF-beta 1, 2 and 3 isotypes in multiple sclerosis. J. Neuroimmunol.

[103] Goris A., Williams-Gray C.H., Foltynie T., Brown J., Maranian M., Walton A., Compston D.A., Barker R.A., Sawcer S.J. (2007). Investigation of TGFB2 as a candidate gene in multiple sclerosis and Parkinson’s disease. J. Neurol.

[104] Keidel M., Stude P, Ramachandran V.S. (2002). Brain Lesions. Encyclopedia of the Human Brain.

[105] Peskind E.R. (1996). Neurobiology of Alzheimer’s disease. J. Clin. Psychiatry.

[106] Caraci F., Battaglia G., Bruno V., Bosco P., Carbonaro V., Giuffrida M.L., Drago F., Sortino M.A., Nicoletti F., Copani A. (2011). TGF-beta1 pathway as a new target for neuroprotection in Alzheimer’s disease. CNS Neurosci. Ther.

[107] Wirths O., Breyhan H., Marcello A., Cotel M.C., Bruck W., Bayer T.A. (2010). Inflammatory changes are tightly associated with neurodegeneration in the brain and spinal cord of the APP/PS1KI mouse model of Alzheimer’s disease. Neurobiol. Aging.

[108] Lippa C.F., Smith T.W., Flanders K.C. (1995). Transforming growth factor-beta: Neuronal and glial expression in CNS degenerative diseases. Neurodegeneration.

[109] Morimoto K., Horio J., Satoh H., Sue L., Beach T., Arita S., Tooyama I., Konishi Y (2011). Expression profiles of cytokines in the brains of Alzheimer’s disease (AD) patients compared to the brains of non-demented patients with and without increasing AD pathology. J. Alzheimers Dis.

[110] Lifshitz V., Weiss R., Benromano T., Kfir E., Blumenfeld-Katzir T., Tempel-Brami C., Assaf Y., Xia W., Wyss-Coray T., Weiner H.L., Frenkel D (2012). Immunotherapy of cerebrovascular amyloidosis in a transgenic mouse model. Neurobiol. Aging.

[111] Uribe-San Martin R., Herrera-Molina R., Olavarria L., Ramirez G., von Bernhardi R. (2009). Reduction of beta-amyloid-induced neurotoxicity on hippocampal cell cultures by moderate acidosis is mediated by transforming growth factor beta. Neuroscience.

[112] Tesseur I., Zou K., Esposito L., Bard F., Berber E., Can J.V., Lin A.H., Crews L., Tremblay P., Mathews P. (2006). Deficiency in neuronal TGF-beta signaling promotes neurodegeneration and Alzheimer’s pathology. J. Clin. Invest.

[113] Rezai-Zadeh K., Gate D., Gowing G., Town T. (2011). How to get from here to there: Macrophage recruitment in Alzheimer’s disease. Curr. Alzheimer. Res.

[114] El Khoury J., Luster A.D. (2008). Mechanisms of microglia accumulation in Alzheimer’s disease: Therapeutic implications. Trends Pharmacol. Sci.

[115] Weiss R., Lifshitz V., Frenkel D. (2011). TGF-beta1 affects endothelial cell interaction with macrophages and T cells leading to the development of cerebrovascular amyloidosis. Brain Behav. Immun.

[116] Wyss-Coray T., Lin C., von Euw D., Masliah E., Mucke L., Lacombe P. (2000). Alzheimer’s disease-like cerebrovascular pathology in transforming growth factor-beta 1 transgenic mice and functional metabolic correlates. Ann. N. Y. Acad. Sci.

[117] Nicolakakis N., Aboulkassim T., Aliaga A., Tong X.K., Rosa-Neto P., Hamel E. (2011). Intact memory in TGF-beta1 transgenic mice featuring chronic cerebrovascular deficit: Recovery with pioglitazone. J. Cereb. Blood Flow Metab.

[118] Ongali B., Nicolakakis N., Lecrux C., Aboulkassim T., Rosa-Neto P., Papadopoulos P., Tong X.K., Hamel E. (2011). Transgenic mice overexpressing APP and transforming growth factor-beta1 feature cognitive and vascular hallmarks of Alzheimer's disease. Am. J. Pathol.

[119] Wyss-Coray T., Lin C., Yan F., Yu G.Q., Rohde M., McConlogue L., Masliah E., Mucke L. (2001). TGF-beta1 promotes microglial amyloid-beta clearance and reduces plaque burden in transgenic mice. Nat. Med.

[120] Caraci F., Bosco P., Signorelli M., Spada R.S., Cosentino F.I., Toscano G., Bonforte C., Muratore S., Prestianni G., Panerai S. (2012). The CC genotype of transforming growth factor-beta1 increases the risk of late-onset Alzheimer’s disease and is associated with AD-related depression. Eur. Neuropsychopharmacol.

[121] Haas A., Liraz O., Michaelson D.M. (2012). The Effects of Apolipoproteins E3 and E4 on the transforming growth factor-beta system in targeted replacement Mice. Neurodegener. Dis.

[122] Lee H.G., Ueda M., Zhu X., Perry G., Smith M.A. (2006). Ectopic expression of phospho-Smad2 in Alzheimer’s disease: Uncoupling of the transforming growth factor-beta pathway?. J. Neurosci. Res.

[123] Ueberham U., Ueberham E., Gruschka H., Arendt T. (2006). Altered subcellular location of phosphorylated Smads in Alzheimer’s disease. Eur. J. Neurosci.

[124] Ueberham U., Hilbrich I., Ueberham E., Rohn S., Glockner P., Dietrich K., Bruckner M.K., Arendt T (2012). Transcriptional control of cell cycle-dependent kinase 4 by Smad proteins-implications for Alzheimer’s disease. Neurobiol. Aging.

[125] Caraci F., Spampinato S., Sortino M.A., Bosco P., Battaglia G., Bruno V., Drago F., Nicoletti F., Copani A. (2012). Dysfunction of TGF-beta1 signaling in Alzheimer’s disease: Perspectives for neuroprotection. Cell Tissue Res.

[126] Harris M.K., Shneyder N., Borazanci A., Korniychuk E., Kelley R.E., Minagar A. (2009). Movement disorders. Med. Clin. North Am.

[127] Krieglstein K., Unsicker K. (1994). Transforming growth factor-beta promotes survival of midbrain dopaminergic neurons and protects them against *N*-methyl-4-phenylpyridinium ion toxicity. Neuroscience.

[128] Schober A., Peterziel H., von Bartheld C.S., Simon H., Krieglstein K., Unsicker K. (2007). GDNF applied to the MPTP-lesioned nigrostriatal system requires TGF-beta for its neuroprotective action. Neurobiol. Dis.

[129] Krieglstein K., Suter-Crazzolara C., Fischer W.H., Unsicker K. (1995). TGF-beta superfamily members promote survival of midbrain dopaminergic neurons and protect them against MPP^+^ toxicity. Embo. J.

[130] Krieglstein K., Suter-Crazzolara C., Unsicker K. (1995). Development of mesencephalic dopaminergic neurons and the transforming growth factor-beta superfamily. J. Neural. Transm. Suppl.

[131] Farkas L.M., Dunker N., Roussa E., Unsicker K., Krieglstein K. (2003). Transforming growth factor-beta(s) are essential for the development of midbrain dopaminergic neurons *in vitro* and *in vivo*. J. Neurosci.

[132] Krieglstein K., Henheik P., Farkas L., Jaszai J., Galter D., Krohn K., Unsicker K. (1998). Glial cell line-derived neurotrophic factor requires transforming growth factor-beta for exerting its full neurotrophic potential on peripheral and CNS neurons. J. Neurosci.

[133] Poulsen K.T., Armanini M.P., Klein R.D., Hynes M.A., Phillips H.S., Rosenthal A. (1994). TGF beta 2 and TGF beta 3 are potent survival factors for midbrain dopaminergic neurons. Neuron.

[134] Roussa E., Oehlke O., Rahhal B., Heermann S., Heidrich S., Wiehle M., Krieglstein K. (2008). Transforming growth factor beta cooperates with persephin for dopaminergic phenotype induction. Stem Cells.

[135] Roussa E., von Bohlen und Halbach O., Krieglstein K. (2009). TGF-beta in dopamine neuron development, maintenance and neuroprotection. Adv. Exp. Med. Biol.

[136] Zhang J., Pho V., Bonasera S.J., Holtzman J., Tang A.T., Hellmuth J., Tang S., Janak P.H., Tecott L.H., Huang E.J. (2007). Essential function of HIPK2 in TGFbeta-dependent survival of midbrain dopamine neurons. Nat. Neurosci.

[137] Polazzi E., Altamira L.E., Eleuteri S., Barbaro R., Casadio C., Contestabile A., Monti B. (2009). Neuroprotection of microglial conditioned medium on 6-hydroxydopamine-induced neuronal death: Role of transforming growth factor beta-2. J. Neurochem.

[138] Rahhal B., Heermann S., Ferdinand A., Rosenbusch J., Rickmann M., Krieglstein K. (2009). *In vivo* requirement of TGF-beta/GDNF cooperativity in mouse development: Focus on the neurotrophic hypothesis. Int. J. Dev. Neurosci.

[139] Tapia-Gonzalez S., Giraldez-Perez R.M., Cuartero M.I., Casarejos M.J., Mena M.A., Wang X.F., Sanchez-Capelo A. (2011). Dopamine and alpha-synuclein dysfunction in Smad3 null mice. Mol. Neurodegener.

[140] Brundin P., Karlsson J., Emgard M., Schierle G.S., Hansson O., Petersen A., Castilho R.F. (2000). Improving the survival of grafted dopaminergic neurons: A review over current approaches. Cell Transplant.

[141] Mantel P.Y., Schmidt-Weber C.B. (2011). Transforming growth factor-beta: Recent advances on its role in immune tolerance. Methods Mol. Biol.

[142] Nishino Y., Ooishi R., Kurokawa S., Fujino K., Murakami M., Madarame H., Hashimoto O., Sugiyama K., Funaba M. (2009). Gene expression of the TGF-beta family in rat brain infected with Borna disease virus. Microbes Infect.

[143] Stitz L., Planz O., Bilzer T., Frei K., Fontana A. (1991). Transforming growth factor-beta modulates T cell-mediated encephalitis caused by Borna disease virus. Pathogenic importance of CD8^+^ cells and suppression of antibody formation. J. Immunol.

[144] Baloul L., Lafon M. (2003). Apoptosis and rabies virus neuroinvasion. Biochimie.

[145] Kossmann T., Morganti-Kossmann M.C., Orenstein J.M., Britt W.J., Wahl S.M., Smith P.D. (2003). Cytomegalovirus production by infected astrocytes correlates with transforming growth factor-beta release. J. Infect. Dis.

[146] Enam S., Sweet T.M., Amini S., Khalili K., del Valle L. (2004). Evidence for involvement of transforming growth factor beta1 signaling pathway in activation of JC virus in human immunodeficiency virus 1-associated progressive multifocal leukoencephalopathy. Arch. Pathol. Lab. Med.

[147] Cupp C., Taylor J.P., Khalili K., Amini S. (1993). Evidence for stimulation of the transforming growth factor beta 1 promoter by HIV-1 Tat in cells derived from CNS. Oncogene.

[148] Sawaya B.E., Thatikunta P., Denisova L., Brady J., Khalili K., Amini S. (1998). Regulation of TNFalpha and TGFbeta-1 gene transcription by HIV-1 Tat in CNS cells. J. Neuroimmunol.

[149] Masliah E., Ge N., Achim C.L., Wiley C.A. (1994). Cytokine receptor alterations during HIV infection in the human central nervous system. Brain Res.

[150] Johnson M.D., Kim P., Tourtellotte W., Federspiel C.F. (2004). Transforming growth factor beta and monocyte chemotactic protein-1 are elevated in cerebrospinal fluid of immunocompromised patients with HIV-1 infection. J. Neuro. AIDS.

[151] da Cunha A., Jefferson J.J., Tyor W.R., Glass J.D., Jannotta F.S., Cottrell J.R., Resau J.H. (1997). Transforming growth factor-beta1 in adult human microglia and its stimulated production by interleukin-1. J. Interferon. Cytokine. Res.

[152] Vitkovic L. (1997). Neuropathogenesis of HIV-1 infection: Interactions between interleukin-1 and transforming growth factor-beta 1. Mol. Psychiatry.

[153] Perrella O., Carreiri P.B., Perrella A., Sbreglia C., Gorga F., Guarnaccia D., Tarantino G. (2001). Transforming growth factor beta-1 and interferon-alpha in the AIDS dementia complex (ADC): Possible relationship with cerebral viral load?. Eur. Cytokine Netw.

[154] Ossege L.M., Voss B., Wiethege T., Sindern E., Malin J.P. (1994). Detection of transforming growth factor beta 1 mRNA in cerebrospinal fluid cells of patients with meningitis by non-radioactive *in situ* hybridization. J. Neurol.

[155] Matsumura S., Shibakusa T., Fujikawa T., Yamada H., Inoue K., Fushiki T. (2007). Increase in transforming growth factor-beta in the brain during infection is related to fever, not depression of spontaneous motor activity. Neuroscience.

[156] Fontana A., Constam D.B., Frei K., Malipiero U., Pfister H.W. (1992). Modulation of the immune response by transforming growth factor beta. Int. Arch. Allergy Immunol.

[157] Malipiero U., Koedel U., Pfister W., Fontana A. (2007). Bacterial meningitis: The role of transforming growth factor-Beta in innate immunity and secondary brain damage. Neurodegener. Dis.

[158] Platten M., Wick W., Weller M. (2001). Malignant glioma biology: Role for TGF-beta in growth, motility, angiogenesis, and immune escape. Microsc. Res. Tech.

[159] Rich J.N., Zhang M., Datto M.B., Bigner D.D., Wang X.F. (1999). Transforming growth factor-beta-mediated p15(INK4B) induction and growth inhibition in astrocytes is SMAD3-dependent and a pathway prominently altered in human glioma cell lines. J. Biol. Chem.

[160] Sasaki A., Naganuma H., Satoh E., Kawataki T., Amagasaki K., Nukui H (2001). Participation of thrombospondin-1 in the activation of latent transforming growth factor-beta in malignant glioma cells. Neurol. Med. Chir. (Tokyo).

[161] Jennings M.T., Pietenpol J.A. (1998). The role of transforming growth factor beta in glioma progression. J. Neurooncol.

[162] Zhang L., Sato E., Amagasaki K., Nakao A., Naganuma H. (2006). Participation of an abnormality in the transforming growth factor-beta signaling pathway in resistance of malignant glioma cells to growth inhibition induced by that factor. J. Neurosurg.

[163] Ikushima H., Todo T., Ino Y., Takahashi M., Miyazawa K., Miyazono K. (2009). Autocrine TGF-beta signaling maintains tumorigenicity of glioma-initiating cells through Sry-related HMG-box factors. Cell Stem Cell.

[164] Hau P., Jachimczak P., Schlaier J., Bogdahn U. (2011). TGF-beta2 signaling in high-grade gliomas. Curr. Pharm. Biotechnol.

[165] Wick W., Naumann U., Weller M. (2006). Transforming growth factor-beta: A molecular target for the future therapy of glioblastoma. Curr. Pharm. Des.

[166] Naumann U., Maass P., Gleske A.K., Aulwurm S., Weller M., Eisele G. (2008). Glioma gene therapy with soluble transforming growth factor-beta receptors II and III. Int. J. Oncol.

[167] Ueda R., Fujita M., Zhu X., Sasaki K., Kastenhuber E.R., Kohanbash G., McDonald H.A., Harper J., Lonning S., Okada H. (2009). Systemic inhibition of transforming growth factor-beta in glioma-bearing mice improves the therapeutic efficacy of glioma-associated antigen peptide vaccines. Clin. Cancer Res.

[168] Kawano H., Kimura-Kuroda J., Komuta Y., Yoshioka N., Li H.P., Kawamura K., Li Y., Raisman G. (2012). Role of the lesion scar in the response to damage and repair of the central nervous system. Cell Tissue Res.

[169] Hunt N.H., Grau G.E. (2003). Cytokines: Accelerators and brakes in the pathogenesis of cerebral malaria. Trends Immunol.

[170] Morganti-Kossmann M.C., Rancan M., Stahel P.F., Kossmann T. (2002). Inflammatory response in acute traumatic brain injury: A double-edged sword. Curr. Opin. Crit. Care.

[171] Dheen S.T., Kaur C., Ling E.A. (2007). Microglial activation and its implications in the brain diseases. Curr. Med. Chem.

[172] Kiefer R., Streit W.J., Toyka K.V., Kreutzberg G.W., Hartung H.P. (1995). Transforming growth factor-beta 1: A lesion-associated cytokine of the nervous system. Int. J. Dev. Neurosci.

[173] Kulkarni A.B., Huh C.G., Becker D., Geiser A., Lyght M., Flanders K.C., Roberts A.B., Sporn M.B., Ward J.M., Karlsson S. (1993). Transforming growth factor beta 1 null mutation in mice causes excessive inflammatory response and early death. Proc. Natl. Acad. Sci. USA.

[174] Wyss-Coray T., Borrow P., Brooker M.J., Mucke L. (1997). Astroglial overproduction of TGF-beta 1 enhances inflammatory central nervous system disease in transgenic mice. J. Neuroimmunol.

[175] Stoll G., Schroeter M., Jander S., Siebert H., Wollrath A., Kleinschnitz C., Bruck W. (2004). Lesion-associated expression of transforming growth factor-beta-2 in the rat nervous system: Evidence for down-regulating the phagocytic activity of microglia and macrophages. Brain Pathol.

[176] Yu P., Wang H., Katagiri Y., Geller H.M. (2012). An *in vitro* model of reactive astrogliosis and its effect on neuronal growth. Method. Mol. Biol.

[177] Johns L.D., Babcock G., Green D., Freedman M., Sriram S., Ransohoff R.M. (1992). Transforming growth factor-beta 1 differentially regulates proliferation and MHC class-II antigen expression in forebrain and brainstem astrocyte primary cultures. Brain Res.

[178] Cullen D.K., Simon C.M., LaPlaca M.C. (2007). Strain rate-dependent induction of reactive astrogliosis and cell death in three-dimensional neuronal-astrocytic co-cultures. Brain Res.

[179] Logan A., Baird A., Berry M. (1999). Decorin attenuates gliotic scar formation in the rat cerebral hemisphere. Exp. Neurol.

[180] Moon L.D., Fawcett J.W. (2001). Reduction in CNS scar formation without concomitant increase in axon regeneration following treatment of adult rat brain with a combination of antibodies to TGFbeta1 and beta2. Eur. J. Neurosci.

[181] Yoshioka N., Kimura-Kuroda J., Saito T., Kawamura K., Hisanaga S., Kawano H. (2011). Small molecule inhibitor of type I transforming growth factor-beta receptor kinase ameliorates the inhibitory milieu in injured brain and promotes regeneration of nigrostriatal dopaminergic axons. J. Neurosci. Res.

[182] Lagord C., Berry M., Logan A. (2002). Expression of TGFbeta 2 but not TGFbeta 1 correlates with the deposition of scar tissue in the lesioned spinal cord. Mol. Cell. Neurosci.

[183] Heinemann U., Kaufer D., Friedman A. (2012). Blood-brain barrier dysfunction, TGFbeta signaling, and astrocyte dysfunction in epilepsy. Glia.

[184] Schachtrup C., Ryu J.K., Helmrick M.J., Vagena E., Galanakis D.K., Degen J.L., Margolis R.U., Akassoglou K. (2010). Fibrinogen triggers astrocyte scar formation by promoting the availability of active TGF-beta after vascular damage. J. Neurosci.

[185] Moreels M., Vandenabeele F., Dumont D., Robben J., Lambrichts I. (2008). Alpha-smooth muscle actin (alpha-SMA) and nestin expression in reactive astrocytes in multiple sclerosis lesions: Potential regulatory role of transforming growth factor-beta 1 (TGF-beta1). Neuropathol. Appl. Neurobiol.

[186] Schwab J.M., Beschorner R., Nguyen T.D., Meyermann R., Schluesener H.J. (2001). Differential cellular accumulation of connective tissue growth factor defines a subset of reactive astrocytes, invading fibroblasts, and endothelial cells following central nervous system injury in rats and humans. J. Neurotrauma.

[187] Yun S.J., Kim M.O., Kim S.O., Park J., Kwon Y.K., Kim I.S., Lee E.H. (2002). Induction of TGF-beta-inducible gene-h3 (betaig-h3) by TGF-beta1 in astrocytes: Implications for astrocyte response to brain injury. Brain Res. Mol. Brain Res.

[188] Law A.K., Gupta D., Levy S., Wallace D.C., McKeon R.J., Buck C.R. (2004). TGF-beta1 induction of the adenine nucleotide translocator 1 in astrocytes occurs through Smads and Sp1 transcription factors. BMC Neurosci.

[189] Chopp M., Li Y. (1996). Apoptosis in focal cerebral ischemia. Acta Neurochir. Suppl.

[190] Deigner H.P., Haberkorn U., Kinscherf R. (2000). Apoptosis modulators in the therapy of neurodegenerative diseases. Expert Opin. Investig. Drugs.

[191] Namura S., Zhu J., Fink K., Endres M., Srinivasan A., Tomaselli K.J., Yuan J., Moskowitz M.A. (1998). Activation and cleavage of caspase-3 in apoptosis induced by experimental cerebral ischemia. J. Neurosci.

[192] Buisson A., Lesne S., Docagne F., Ali C., Nicole O., MacKenzie E.T., Vivien D. (2003). Transforming growth factor-beta and ischemic brain injury. Cell. Mol. Neurobiol.

[193] Zhu Y., Ahlemeyer B., Bauerbach E., Krieglstein J. (2001). TGF-beta1 inhibits caspase-3 activation and neuronal apoptosis in rat hippocampal cultures. Neurochem. Int.

[194] Bye N., Zieba M., Wreford N.G., Nichols N.R. (2001). Resistance of the dentate gyrus to induced apoptosis during ageing is associated with increases in transforming growth factor-beta1 messenger RNA. Neuroscience.

[195] Zhu Y., Yang G.Y., Ahlemeyer B., Pang L., Che X.M., Culmsee C., Klumpp S., Krieglstein J. (2002). Transforming growth factor-beta 1 increases bad phosphorylation and protects neurons against damage. J. Neurosci.

[196] Ferrer I. (2006). Apoptosis: Future targets for neuroprotective strategies. Cerebrovasc. Dis.

[197] Kim E.S., Kim R.S., Ren R.F., Hawver D.B., Flanders K.C. (1998). Transforming growth factor-beta inhibits apoptosis induced by beta-amyloid peptide fragment 25–35 in cultured neuronal cells. Brain Res. Mol. Brain Res.

[198] Caraci F., Battaglia G., Busceti C., Biagioni F., Mastroiacovo F., Bosco P., Drago F., Nicoletti F., Sortino M.A., Copani A. (2008). TGF-beta 1 protects against Abeta-neurotoxicity via the phosphatidylinositol-3-kinase pathway. Neurobiol. Dis.

[199] Hicks S.D., Miller M.W. (2011). Effects of ethanol on transforming growth factor Beta1-dependent and-independent mechanisms of neural stem cell apoptosis. Exp. Neurol.

[200] Marushige K., Marushige Y. (1994). Induction of apoptosis by transforming growth factor beta 1 in glioma and trigeminal neurinoma cells. Anticancer Res.

[201] Schulz R., Vogel T., Mashima T., Tsuruo T., Krieglstein K. (2009). Involvement of fractin in TGF-beta-induced apoptosis in oligodendroglial progenitor cells. Glia.

[202] Xiao B.G., Bai X.F., Zhang G.X., Link H. (1997). Transforming growth factor-beta1 induces apoptosis of rat microglia without relation to bcl-2 oncoprotein expression. Neurosci. Lett.

[203] Jung B., Kim M.O., Yun S.J., Lee E.H. (2003). Down-regulation of the expression of rat inhibitor-of-apoptosis protein-1 and −3 during transforming growth factor-beta1-mediated apoptosis in rat brain microglia. Neuroreport.

[204] Dunker N., Schmitt K., Schuster N., Krieglstein K. (2002). The role of transforming growth factor beta-2, beta-3 in mediating apoptosis in the murine intestinal mucosa. Gastroenterology.

[205] Prehn J.H., Backhauss C., Krieglstein J. (1993). Transforming growth factor-beta 1 prevents glutamate neurotoxicity in rat neocortical cultures and protects mouse neocortex from ischemic injury *in vivo*. J. Cereb. Blood Flow Metab.

[206] Ho T.W., Bristol L.A., Coccia C., Li Y., Milbrandt J., Johnson E., Jin L., Bar-Peled O., Griffin J.W., Rothstein J.D. (2000). TGFbeta trophic factors differentially modulate motor axon outgrowth and protection from excitotoxicity. Exp. Neurol.

[207] Docagne F., Nicole O., Gabriel C., Fernandez-Monreal M., Lesne S., Ali C., Plawinski L., Carmeliet P., MacKenzie E.T., Buisson A., Vivien D. (2002). Smad3-dependent induction of plasminogen activator inhibitor-1 in astrocytes mediates neuroprotective activity of transforming growth factor-beta 1 against NMDA-induced necrosis. Mol. Cell. Neurosci.

[208] Mesples B., Fontaine R.H., Lelievre V., Launay J.M., Gressens P. (2005). Neuronal TGF-beta1 mediates IL-9/mast cell interaction and exacerbates excitotoxicity in newborn mice. Neurobiol. Dis.

[209] Prehn J.H., Peruche B., Unsicker K., Krieglstein J. (1993). Isoform-specific effects of transforming growth factors-beta on degeneration of primary neuronal cultures induced by cytotoxic hypoxia or glutamate. J. Neurochem.

[210] Kane C.J., Brown G.J., Phelan K.D. (1996). Transforming growth factor-beta2 increases NMDA receptor-mediated excitotoxicity in rat cerebral cortical neurons independently of glia. Neurosci. Lett.

[211] Bae J.J., Xiang Y.Y., Martinez-Canabal A., Frankland P.W., Yang B.B., Lu W.Y. (2011). Increased transforming growth factor-beta1 modulates glutamate receptor expression in the hippocampus. Int. J. Physiol. Pathophysiol. Pharmacol.

[212] Roberts A.B., Sporn M.B., Assoian R.K., Smith J.M., Roche N.S., Wakefield L.M., Heine U.I., Liotta L.A., Falanga V., Kehrl J.H. (1986). Transforming growth factor type beta: Rapid induction of fibrosis and angiogenesis *in vivo* and stimulation of collagen formation *in vitro*. Proc. Natl. Acad. Sci. USA.

[213] Pepper M.S., Vassalli J.D., Orci L., Montesano R. (1993). Biphasic effect of transforming growth factor-beta 1 on *in vitro* angiogenesis. Exp. Cell. Res.

[214] Gajdusek C.M., Luo Z., Mayberg M.R. (1993). Basic fibroblast growth factor and transforming growth factor beta-1: Synergistic mediators of angiogenesis *in vitro*. J. Cell. Physiol.

[215] Fajardo L.F., Prionas S.D., Kwan H.H., Kowalski J., Allison A.C. (1996). Transforming growth factor beta1 induces angiogenesis *in vivo* with a threshold pattern. Lab. Invest.

[216] Bertolino P., Deckers M., Lebrin F., ten Dijke P. (2005). Transforming growth factor-beta signal transduction in angiogenesis and vascular disorders. Chest.

[217] Ferrari G., Cook B.D., Terushkin V., Pintucci G., Mignatti P. (2009). Transforming growth factor-beta 1 (TGF-beta1) induces angiogenesis through vascular endothelial growth factor (VEGF)-mediated apoptosis. J. Cell. Physiol.

[218] Ueki N., Nakazato M., Ohkawa T., Ikeda T., Amuro Y., Hada T., Higashino K. (1992). Excessive production of transforming growth-factor beta 1 can play an important role in the development of tumorigenesis by its action for angiogenesis: Validity of neutralizing antibodies to block tumor growth. Biochim. Biophys. Acta.

[219] Abe K., Chu P.J., Ishihara A., Saito H. (1996). Transforming growth factor-beta 1 promotes re-elongation of injured axons of cultured rat hippocampal neurons. Brain Res.

[220] Yu G., Fahnestock M. (2002). Differential expression of nerve growth factor transcripts in glia and neurons and their regulation by transforming growth factor-beta1. Brain Res. Mol. Brain Res.

[221] Ferrer I., Lopez E., Pozas E., Ballabriga J., Marti E. (1998). Multiple neurotrophic signals converge in surviving CA1 neurons of the gerbil hippocampus following transient forebrain ischemia. J. Comp. Neurol.

